# DA-Transformer: Building Damage Assessment Under Complex Changing Conditions Through Multimodal UAV Data

**DOI:** 10.3390/s26206470

**Published:** 2026-10-13

**Authors:** Zhuoyue Yang, Yuxin Xu, Qizhi Xu, Kang Yu, Hao Song

**Affiliations:** School of Mechatronics Engineering, Beijing Institute of Technology, Beijing 100081, China; yangzhy@bit.edu.cn (Z.Y.); qizhi@bit.edu.cn (Q.X.); 3220240206@bit.edu.cn (K.Y.); 3220240185@bit.edu.cn (H.S.)

**Keywords:** damage assessment, multimodal data, point cloud registration, large language model, view generation

## Abstract

Accurate damage assessment is critical for reconnaissance and rescue missions. Damage assessment based on unmanned aerial vehicle images is a relevant topic. However, sharing pre- and post-damage knowledge is hindered by the lack of same-viewpoint images in damage assessment for building restoration and reconstruction work. We propose a novel method for generating images based on the same view before and after damage occurs. A point cloud registration method based on serialized matching is presented for spatial transformation with complex damage variations, achieving lower errors and reducing training time. Then, the transformed data are utilized to train a pre-damage differentiable rendering model, generating the undamaged images of buildings from post-damage drone views. Therefore, damage assessment using a large language model (LLM) is conducted by comparing pre- and post-damage images and point clouds. We also construct pre- and post-destruction datasets based on videos. Our method overcomes the limitation of obtaining paired image data from the same perspective, improving the efficiency of evaluation and reducing the difficulty and cost of data collection. We validated the effectiveness of the method through scaling experiments and real explosion scenarios.

## 1. Introduction

Building damage assessment is crucial for reconnaissance and post-disaster housing recovery, serving as the core basis for disaster evaluation and the reconstruction of residential areas [1,2]. This assessment typically comprises two sequential phases: the first phase is the emergency response period, during which aerial remote sensing platforms are utilized to conduct rapid disaster reconnaissance of the entire affected area from a bird’s-eye view, focusing on identifying visible structural damage such as collapsed roofs and destroyed bridges to support the urgent formulation of emergency rescue plans. The second phase is the recovery and reconstruction period, during which the assessment of unsafe structures must be carried out, such as residential buildings, bridges, reservoirs, dams, etc. The assessment of the second phase is directly linked to the safety of rescue workers and the safeguarding of essential public infrastructure. The accuracy requirements in this phase are significantly higher. To achieve high precision, some researchers have used displacement sensors to measure deformation [3,4]. Sensor deployment is time-consuming and costly. Mainstream damage assessment methods focus on the first stage of global investigation and rely on satellite remote sensing images. These methods aim to improve the accuracy of building damage classification by extracting changes in the two-dimensional shape and texture of targets within remote sensing images. Satellite imagery is constrained by fixed orbital trajectories and near-vertical viewing geometry, which predominantly reveal roof-level damage. In contrast, unmanned aerial vehicles (UAVs) offer significant advantages in timely assessment, meeting the multi-perspective needs of recovery and reconstruction applications. Therefore, UAV image processing technology holds indispensable research value for the development of damage assessment and structural health detection.

**Figure 1:**
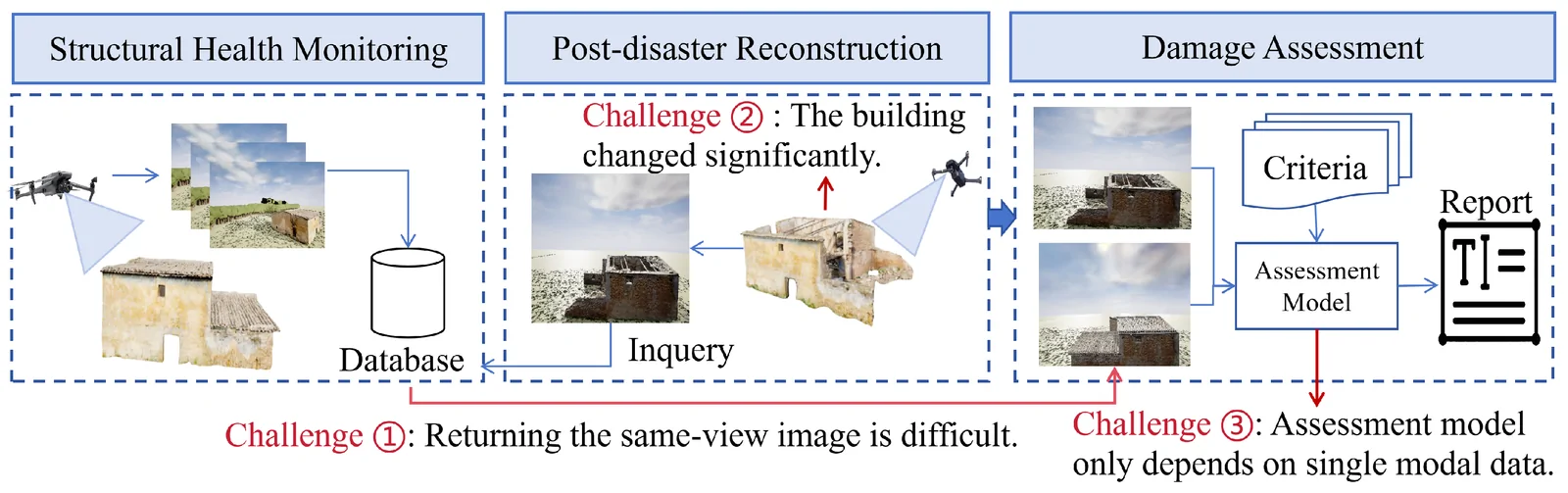
Pipeline of damage assessment based on pre- and post-damage UAV images.

Unlike damage assessment methods based on remote sensing imagery, those based on UAV imagery still face several challenges: (1) UAVs do not follow fixed trajectories, making it difficult to obtain before-and-after images from the same location and perspective. (2) The significant differences and low overlap between undamaged and damaged buildings make spatial registration difficult with existing methods. (3) Building damage assessment methods rely solely on single-modal image data. When images are affected by uncertainties such as insufficient visibility or the presence of smoke, damage level assessments are prone to errors. Figure 1 clearly illustrates these challenges and the damage assessment pipeline based on UAV imagery. Engineers operate UAVs during routine health monitoring activities to acquire images and store them in a database. After damage occurs, rescue personnel use UAVs to collect images of the building and search the database for images from the same angle. Finally, damage assessment is performed by comparing UAV images with the same perspective according to preset criteria. However, due to the lack of images from the same viewpoint, sharing before-and-after data is difficult.

At present, with the development of deep learning, change detection technology based on two-dimensional images has rapidly advanced. Sun et al. [5] used convolution and LSTM as the network backbones for change detection. The self-supervised learning framework and spatial analysis rules were utilized in [6] to improve the accuracy and efficiency of the prediction of 2D building changes. Chen et al. [7] made use of a Transformer backbone to enhance the correlation between buildings in a supervised manner. Semantic information was employed as prior knowledge in [8]. Lin et al. [9] used pseudo-transitional videos and temporal encoders to prevent information loss, and they addressed the difficulty of extracting spatiotemporal features from remote sensing images by utilizing video understanding techniques. Facing the practical demand of improving generalization, Fang et al. [10] aggregated high-resolution features based on a Siamese structure. However, change detection methods based on remote sensing images often rely on obtaining comparative images taken at the same location using fixed satellite trajectories. These methods can not handle images acquired by UAVs with flexible flight trajectories.

There are three reasons why it is difficult to obtain images from the same perspective before and after damage occurs. Firstly, the position and posture of drones used to collect images during health monitoring activities are rarely recorded. Secondly, due to uncertain factors such as excessive wind speed and smoke-induced low visibility, it is also challenging to ensure that the angle and position of the images taken by the drone before and after the damage remain consistent. Obtaining paired images may require a large number of attempts, resulting in increased acquisition costs. Finally, buildings undergo complex and drastic changes after disasters, resulting in inaccurate registration of pre- and post-damage images. The damage assessment method based on image comparison includes steps such as image preprocessing, image registration, and change detection. Two-dimensional image registration methods are based on SIFT [11] and SURF [12] features and have poor robustness to occlusion, noise, and illumination changes, making data preprocessing complex and directly affecting the accuracy of damage assessment. Point cloud registration relies on the results of 3D reconstruction from multi-view images, thereby reducing the dependence on the quality of single-view images. Moreover, registering pre- and post-damage 3D reconstructions primarily exploits the geometric consistency of the point clouds. Therefore, we propose a method for generating images from the same viewpoint based on point cloud registration.

3D models typically refer to point clouds [13] and can be obtained using radar [14] and unmanned aerial vehicle (UAV) photography. Point cloud registration methods often rely on local feature extraction, including statistical and deep learning features. However, these features need to remain unchanged. Charles et al. [15] proposed the PointNet network for directly processing point cloud data by stacking multi-layer perceptron (MLP) modules. Some methods focus on correspondence generation. Yao et al. [16] found the correspondences between point clouds with severe outliers based on the global-to-local research. Yao et al. [17] presented a rotation-equivariant feature extraction network and a hypothesis generation. However, buildings change significantly before and after a disaster, and matching methods relying on local features may not achieve good results. Therefore, improving the performance of point cloud registration methods under conditions of great change is a problem that needs to be solved. We present a serialized matching module for searching for a relative relationship between points.

**Figure 2:**
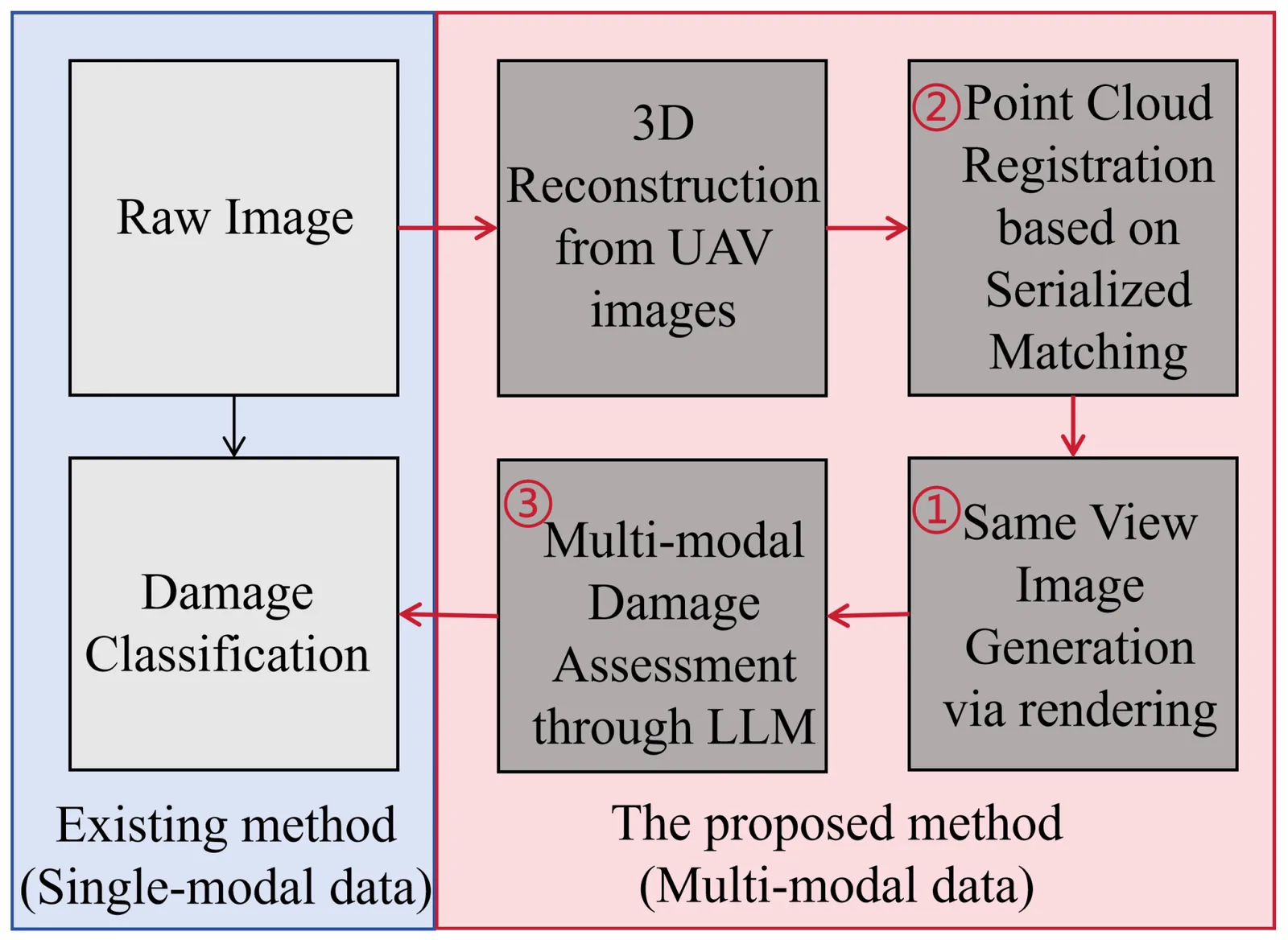
Comparison of existing damage assessment methods and the proposed method.

In addition, damage assessment methods can be divided into four categories: manual judgment [18], fuzzy comprehensive method [19], knowledge-based reasoning [20], and image comparison-based evaluation [21]. Both manual judgment and fuzzy comprehensive evaluation methods rely on subjective judgment. Knowledge-based reasoning methods rely on manually constructed knowledge, which may be derived from labeled image data or domain expert rules, to achieve accurate and real-time reasoning. Image comparison-based damage assessment methods are intuitive, effective, and easy to quantify. However, images from the same viewpoint are difficult to obtain. We combine image comparison methods and a large language model (LLM) to construct a damage assessment method based on multimodal data, as shown in Figure 2.

Therefore, we present a novel method to generate the same view in pre-damage images according to the post-damage UAV images. We generate 3D models before and after damage from drone images, and we utilize 3D point cloud registration to complete the spatial transformation before and after damage. These multimodal data are utilized in the large language model for damage assessment. The main contributions of this paper are as follows:

- We propose a novel method for generating images from the same perspective before and after the damage occurs. Our method overcomes the limitation of obtaining paired image data from the same perspective and reduces the difficulty and cost of data collection.
- A damage assessment method based on multimodal data and a large language model is presented. By comparing 3D models and 2D images, the proposed method can reflect both significant changes (collapsed houses) and details of damage (wall cracks).
- We present a point cloud registration strategy based on serialized matching and deep learning feature extraction. Our method achieves lower errors and reduces the time cost of the training stage.
- We establish a drone image dataset containing buildings with different degrees of damage. We conduct qualitative and quantitative experiments using this dataset. We also validate the effectiveness of the method using scaled models and real explosion scenarios.

The rest of this article is organized as follows. We summarize the related work in Section 2. The proposed framework and point cloud registration methods are described in detail in Section 3. Section 4 shows both quantitative and qualitative experimental results. The discussion is provided in Section 5. Finally, conclusions are drawn in Section 6.

## 2. Related Work

### 2.1. Damage Assessment

Damage assessment for buildings is developing rapidly, undergoing a transition from manual evaluation [18] to automatic evaluation [6]. The development of machine learning and deep learning has brought many opportunities to this field, such as damage level classification based on machine learning [2] and crack detection based on deep learning [22]. Damage assessment methods based on 2D remote sensing images remain an active research area, but most of the existing literature is based on pre-built datasets. Large-scale datasets require long-term data accumulation.

Some researchers have developed assessment methods based on 3D point clouds. Authors [23,24,25] demonstrated that 3D reconstruction from UAV images can be used to monitor the natural gas pipeline infrastructure, a bridge, or a scaled-down dam model. Structure from Motion (SfM) is commonly utilized in 3D reconstruction. Cao et al. [26] enhanced the feature extraction performance of binocular image pairs based on contrastive learning theory and the feature selection step. Many researchers have attempted to utilize CNNs and Transformers for the 3D reconstruction of UAV images [27]. Kim et al. [28] replaced the original image with the image segmentation result and then fed the image into the improved NeRF to obtain a 3D reconstruction result with damage mapping.

### 2.2. Point Cloud Registration

Point cloud registration is an important step. Point cloud registration methods based on deep learning have developed rapidly in recent years and require 3D data to train the model. Coarse registration roughly aligns point clouds with relatively large pose variation, while fine registration usually focuses on minimizing small residual errors [29]. Typical coarse registration methods rely on handcrafted descriptors such as fast point feature histograms (FPFH) [30], etc. Fine registration includes iterative closest point (ICP) [31]. As for deep learning-based methods, Huang et al. [32] proposed a multimodal descriptor for point cloud registration. Chen et al. [33] proposed a misaligned registration network for low-overlap point cloud pairs. Mengiste et al. [34] utilized geometric and position assets to register point clouds and BIMs. Yu et al. [35] used the cross-attention for point correspondences generation. Qin et al. [36] applied the self-attention and cross-attention in feature matching and replaced the RANSAC with the transformation selection operations. From 2023, Yang et al. [37] and Zhang et al. [38] mined more local correspondences in a graph structure.

### 2.3. Novel View Synthesis

The basic form of the Neural Radiance Fields (NeRF) [39] model represents a 3D scene as a radiance field approximated by a neural network, which describes the color and volume density of each point in the scene and each viewing direction. The input of the network is the 3D position and 2D viewing direction, and its output is the color and volume density. Kerbl et al. [40] represent the scene with 3D Gaussians to save computation space.

## 3. Materials and Methods

### 3.1. Overview

**Figure 3:**
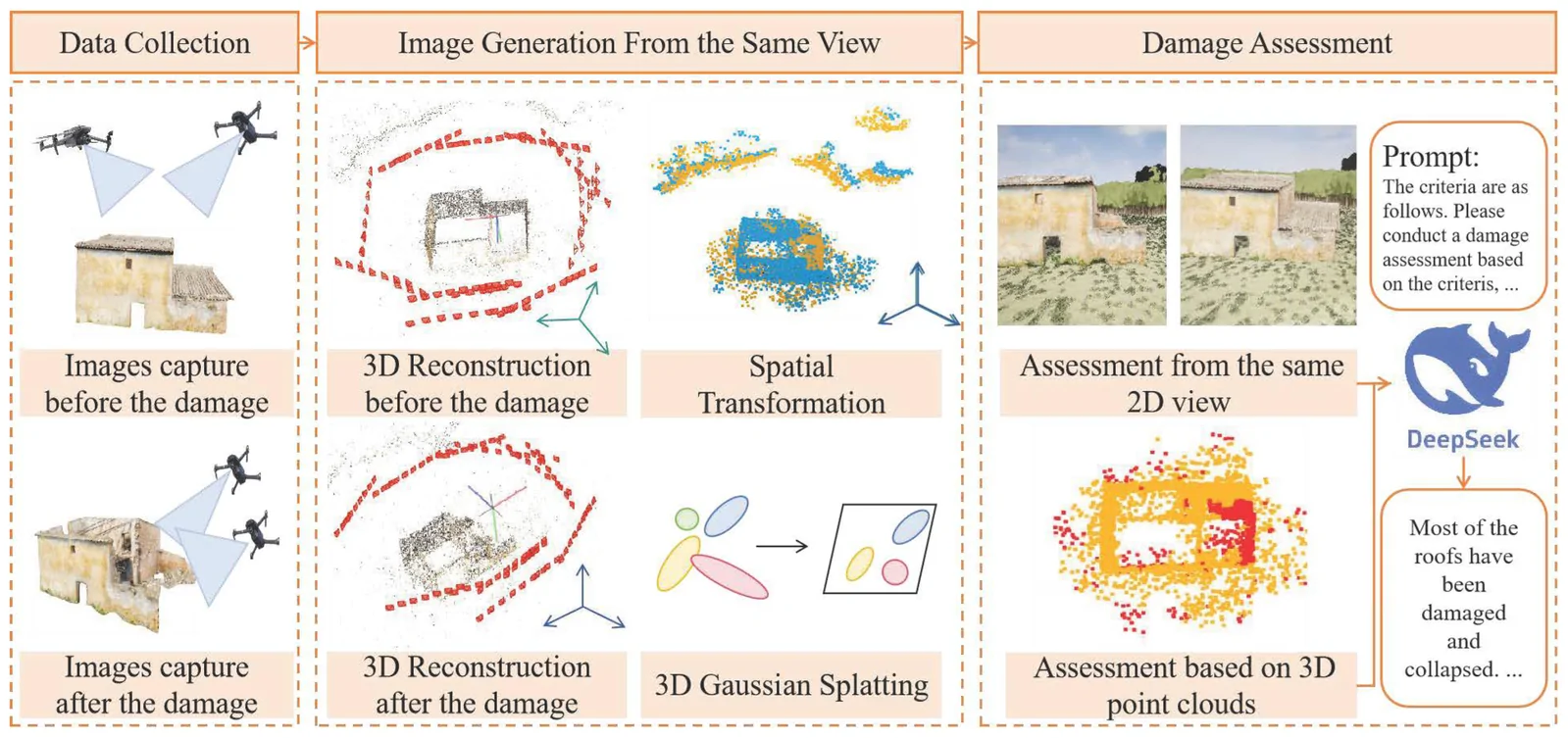
The framework of our proposed method. Orange arrows represent the direction of data flow, and blue intersecting arrows represent coordinate systems.

The framework is shown in Figure 3. Our framework consists of three parts: data collection, same-view image generation, and damage assessment. A drone is used to capture images of the building before and after the incident. According to UAV images captured before the damage, a pre-damage multi-view image dataset is constructed by the structure from motion (SfM) method. The dataset includes images ($I_{o}$), poses ($P_{o}$), and the point cloud ($C_{o}$) before damage occurs.

A post-damage multi-view UAV image data set is obtained using the SfM method. And, the dataset includes images ($I_{e}$), poses ($P_{e}$), and the point cloud ($C_{e}$) of the damaged buildings. The point cloud $C_{o}$ and the damaged point cloud $C_{e}$ are used for point cloud registration (*R*) to obtain the transformation matrix (T) representing rotation and translation. Through the transformation matrix, the pre-damage image pose data and the pre-damage point cloud data are converted to the post-damage point cloud space. After the spatial transformation (*S*), the pre-damage image pose data is marked as $P_{t}$, and the pre-damage point cloud data is marked as $C_{t}$. The pre-damage image pose data, the pre-damage point cloud, and the pre-damage image data after the spatial conversion are used as inputs, and the pre-damage model is obtained by training a differentiable renderer (*G*). The post-damage image pose data ($P_{e}$) is used as input, and the pre-damage image corresponding to the post-damage perspective is generated by the differentiable renderer according to the pre-damage model (M). The damage assessment (*D*) is performed through the pre-damage images ($I_{g}$) and post-damage images ($I_{e}$) of the same perspective, the pre-damage point cloud ($C_{t}$), and the post-damage point cloud ($C_{e}$). The overall process is shown in Algorithm 1. 
**Algorithm 1** Same view image generation and damage assessment**Input:** UAV images before the damage $I_{o}$ and UAV images after the damage $I_{e}$ (No need for images from the same perspective); **Output:** Damage assessment of the building D;
1:$C_{o},P_{o}\leftarrow SfM\left(I_{o}\right)$2:$C_{e},P_{e}\leftarrow SfM\left(I_{e}\right)$3:$T\leftarrow R(C_{o},C_{e})$4:$C_{t},P_{t}\leftarrow S\left(C_{o},P_{o},T\right)$5:$M\leftarrow G(C_{t},P_{t},I_{o})$6:$I_{g}\leftarrow Render\left(M,P_{e}\right)$7:$D\leftarrow D(\left(I_{g},I_{e}\right),\left(C_{t},C_{e}\right))$8:**return D**.


For 3D reconstruction, a drone is utilized to collect images of the damaged building. The dataset constructed prior to the damage comprises multi-perspective images of the building’s front, rear, left, right, and top. The images captured by the drone after the damage do not need to be taken from the same perspective as before. These images are used as input by the structure from motion method [41] to obtain the image pose and the point cloud data. This approach resolves the issue of unsynchronized recording of captured images and drone poses during building health inspection activities.

### 3.2. Point Cloud Registration via Serialized Matching

The purpose of this paper is to restore the image from the same perspective, using the camera pose obtained after the damage. The spatial transformation is achieved through point cloud registration. The result of point cloud registration is a transformation matrix. All data items (the point cloud, camera poses) before the damage need to be spatially transformed through this transformation matrix. Accurate point cloud registration results are crucial for spatial transformation. The core challenge lies in registering the partial point cloud after damage with the complete model prior to damage. Having more points does not necessarily lead to more accurate correspondences. Most methods use uniform sampling as a preprocessing step. Our analysis revealed that the edges of key structures play a crucial role in point cloud registration.

**Figure 4:**
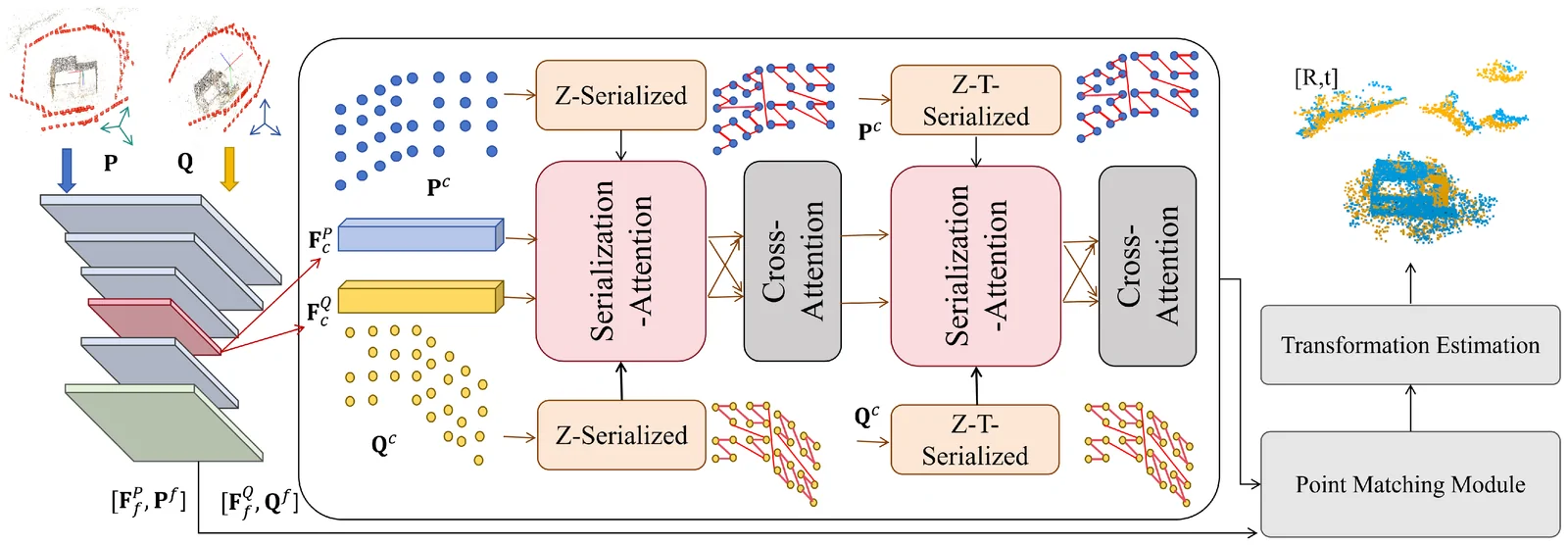
The point cloud registration method based on the serialized matching. Arrows represent the data flow, and the pink boxes denote our proposed serialized attention module.

We present a serialization attention-based point cloud registration algorithm, as shown in Figure 4. The point clouds P and Q are the input of point cloud registration. The multi-level features are extracted from the KPConv-FPN [42] backbone. The associated points and learned features corresponding to the coarsest resolution are $P^{c}$ and $F_{c}^{P}$. The associated points and learned features corresponding to the finest resolution are $P^{f}$ and $F_{f}^{P}$. The downsampled point clouds of Q are denoted as $Q^{c}$ and $Q^{f}$. $F_{c}^{Q}$$F_{f}^{Q}$ represents the coarsest point cloud feature and the finest feature of Q, respectively. The coarsest point clouds $P^{c}$ and $Q^{c}$ are serialized with z-order curve. The serialized attention module and cross-attention module are interleaved for two times. The learned feature $F_{c}^{P}$ and serialized point cloud $P^{c}$ with mapping function $\psi_{z}$ are fed into the serialized Attention module and generates result ${\tilde{F}}_{Self_{z}}^{P}$ in Equation (1). The output of serialization attention is put forward into the cross attention module in Equation (2). The result of cross attention module in first iteration is defined as ${\tilde{F}}_{Cross_{z}}^{P}$ and ${\tilde{F}}_{Cross_{z}}^{Q}$.

(1) $$\begin{array}{c} {\tilde{F}}_{Self_{z}}^{P}=SerializedAtten\left(P^{c},\psi_{z},F_{c}^{P}\right),\\ {\tilde{F}}_{Self_{z}}^{Q}=SerializedAtten\left(Q^{c},\psi_{z},F_{c}^{Q}\right) \end{array}$$

(2) $$\begin{array}{c} {\tilde{F}}_{Cross_{z}}^{P}=CrossAtten\left({\tilde{F}}_{Self_{z}}^{P},{\tilde{F}}_{Self_{z}}^{Q}\right),\\ {\tilde{F}}_{Cross_{z}}^{Q}=CrossAtten\left({\tilde{F}}_{Self_{z}}^{Q},{\tilde{F}}_{Self_{z}}^{P}\right) \end{array}$$

**Figure 5:**
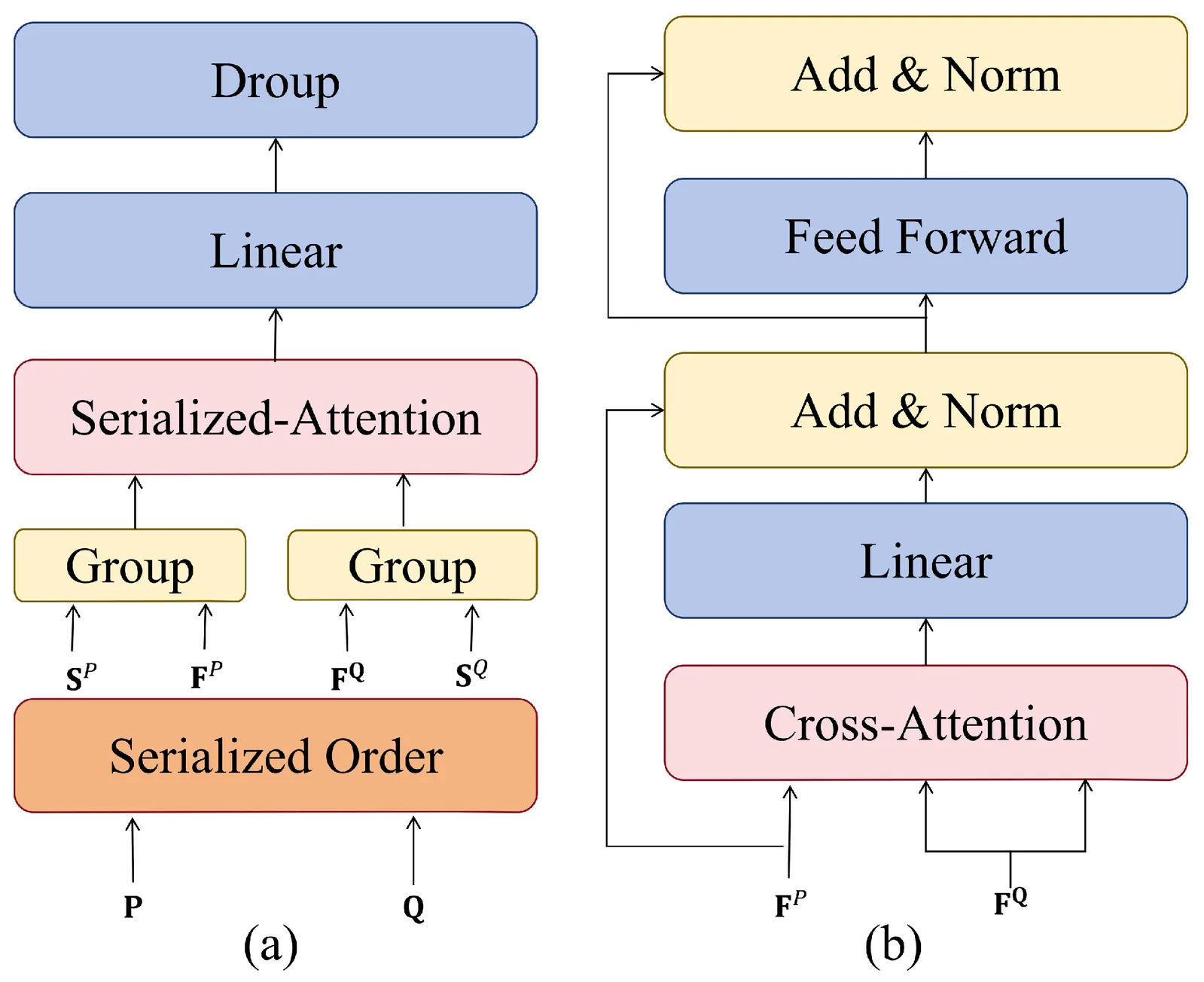
The structure of serialized attention and cross-attention module. (**a**) serialized attention module. (**b**) cross attention module.

At the next step, $P^{c}$ and $Q^{c}$ are serialized with z-trans-order curve [43]. The ${\tilde{F}}_{Cross_{z}}^{P}$ and ${\tilde{F}}_{Cross_{z}}^{Q}$ are served as the input of the second iteration, as shown in Equations (3) and (4). The details of each module are shown in Figure 5.

(3) $$\begin{array}{c} {\tilde{F}}_{Self_{z-t}}^{P}=SerializedAtten\left(P^{c},\psi_{z-t},{\tilde{F}}_{Cross_{z}}^{P}\right),\\ {\tilde{F}}_{Self_{z-t}}^{Q}=SerializedAtten\left(Q^{c},\psi_{z-t},{\tilde{F}}_{Cross_{z}}^{Q}\right) \end{array}$$

(4) $$\begin{array}{c} {\tilde{F}}_{Cross_{z-t}}^{P}=CrossAtten\left({\tilde{F}}_{Self_{z-t}}^{P},{\tilde{F}}_{Self_{z-t}}^{Q}\right),\\ {\tilde{F}}_{Cross_{z-t}}^{Q}=CrossAtten\left({\tilde{F}}_{Self_{z-t}}^{Q},{\tilde{F}}_{Self_{z-t}}^{P}\right) \end{array}$$

We utilize the serialized matching to encode the intra-point-cloud geometric structure and high-level features. The point relationship is embedded in the space-filling curves. The serialized matching module consists of serialization embedding and serialized-attention operation.

The serialized attention module is shown in Figure 5a. The encoder includes grid pooling, shuffle orders and Layer Normalization (LN). Convolution-based methods rely on extracting features from neighboring points, but such features focus solely on local geometric structures. We utilize the serialized encoding strategy through space-filling curves, as shown in Equation (5). They group points into non-overlapping patches and perform attention within each individual patch. The learned feature F are acquired from the 1/8 sampled points p. As shown in Equation (6), the serialized feature $\hat{F}$ is acquired according to the mapping function γ and F.

(5) γ(p)=(b<<k)|(⌊p/g⌋)

(6) $$\hat{F}=F〚\gamma (p)〛$$

The input feature F is projected to the same dimensional queries $Q=\hat{F}W_{q}$, keys $K=\hat{F}W_{k}$, values $V=\hat{F}W_{v}$, where $W_{q}$, $W_{k}$, $W_{v}$ are weight matrices. The serialized attention operation is shown as follows:

(7) $$\tilde{F}=\mathrm{Softmax}\left(\frac{QK^{⊤}}{\sqrt{d_{t}}}\right)V+\hat{F}.$$

The cross attention module is shown in Figure 5b. The cross-attention module is widely used in feature-based similarity computation. Considering the serialized feature $F_{self}^{P}$ and $F_{s}^{Q}$ acquired from the serialized attention module, our cross-attention module output $F_{cross}^{P}$ as follows:

(8) $${\bar{F}}_{cross}^{P}=F_{self}^{P}+Linear\left(LN\left(crossAtten\left(F_{self}^{P},F_{self}^{Q}\right)\right)\right),$$

where crossAtten represents dot-attention, which is the same as Equation (7), and LN represents a layer normalization operation. Then, the non-linearity of the features can be increased:

(9) $$F_{cross}^{P}={\bar{F}}_{self}^{P}+Linear\left(FFN\left({\bar{F}}_{self}^{P}\right)\right),$$

where FFN is an abbreviation for feedforward neural network. Through sequential point matching, our method extends the field of view for feature extraction, thereby effectively leveraging the geometric adjacency relationships between coarse points.

### 3.3. Same View Generation Using 3D Gaussian Splatting

Although novel view synthesis is a popular area of research, most studies focus on generating images from unseen perspectives based on images captured at the same time and from the same position. In contrast, our approach is centered on scenarios involving long observation intervals, during which time complex changes occur. Rendering methods have developed rapidly in recent years. The main disadvantages of the neural radiance field are its slow rendering speed and poor generalization ability. 3D Gaussian splatting [40] is a real-time rendering method proposed in 2023. This method can achieve state-of-the-art visual effects while maintaining competitive training time. This ensures the timeliness of the proposed method.

In our method, the spatially transformed point clouds and images are utilized to train the 3D Gaussian model. A 3D Gaussian includes a center position μ, opacity α, a 3D covariance matrix ∑, and a color c. The color c is represented by a spherical harmonic function to achieve a view-dependent appearance display. The Gaussian splatting technique projects the pre-damaged 3D Gaussian points into a 2D picture for rendering. Given the observation transformation W and the 3D covariance matrix ∑, the projected 2D covariance matrix $\sum^{\prime }$ is calculated. The transformed images are used as ground truth during the training process. In summary, we constructed a 3D Gaussian model of the complete building in the post-damage environment space.

### 3.4. Damage Assessment Based on LLM

**Figure 6:**
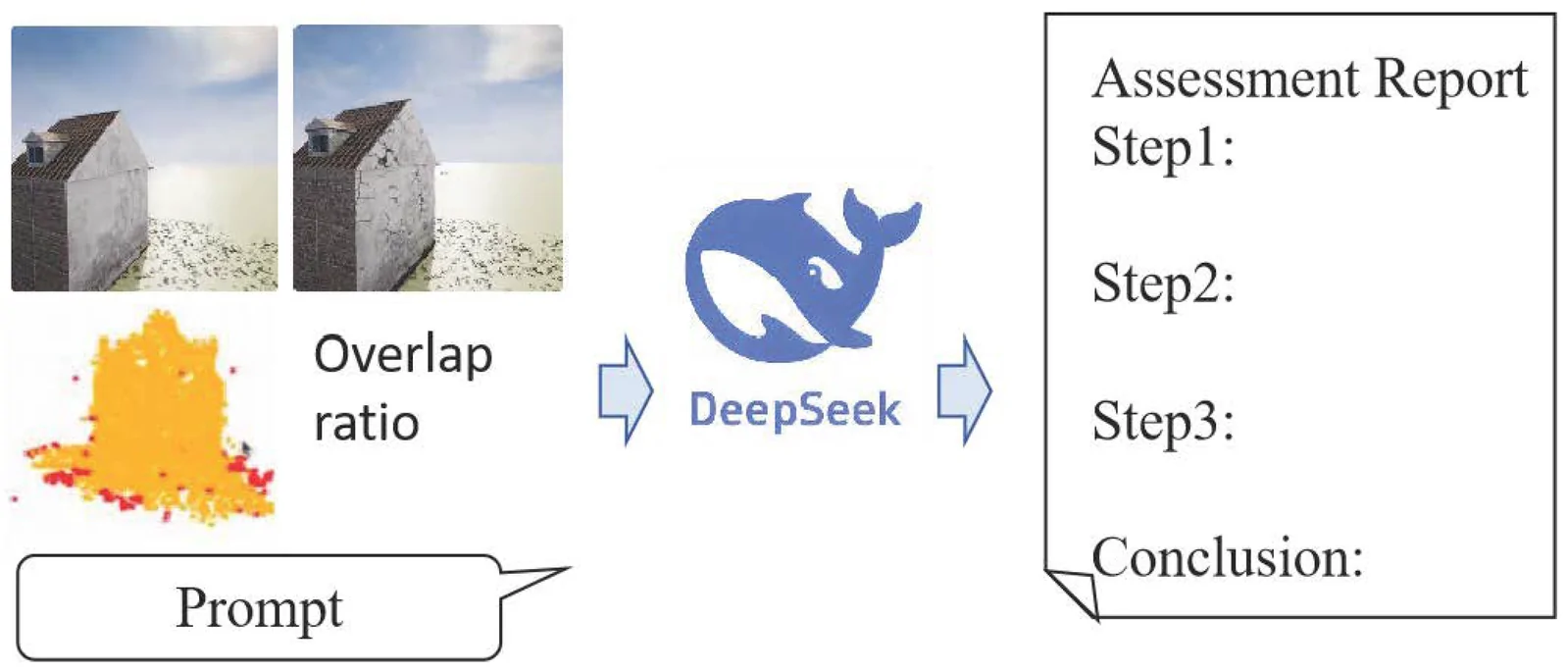
Schematic diagram of Damage assessment based on LLM. The red dots represent the changed 3D points, and the yellow dots represent the unchanged 3D points.

Drones can discover several kinds of damaged buildings with collapsed roofs or wall cracks using our proposed method. The method is shown in Figure 6. We utilized the LLM model DeepSeek [44]. The recovered pre-damage images, post-damage images, the overlap ratio, and the heat map of the change area after point cloud registration are utilized as the input. Through carefully designed prompt words, our proposed method can be applied to the damage assessment frameworks of different countries by quantifying the damage. Different countries seldom have the same standards for the degree of damage, which can be divided into 3, 4, or 5 categories. Therefore, this article focuses on the technical methods of change detection rather than directly associating images with damage levels.

For buildings that have not collapsed, our proposed method reflects the damage by comparing two-dimensional images from the same perspective (crack detection). For severely damaged structures, our proposed method can assess the 3D damage condition by measuring the overlap rate between the post-damage point cloud and the pre-damage point cloud.

## 4. Results

### 4.1. Data Preparation

**Figure 7:**
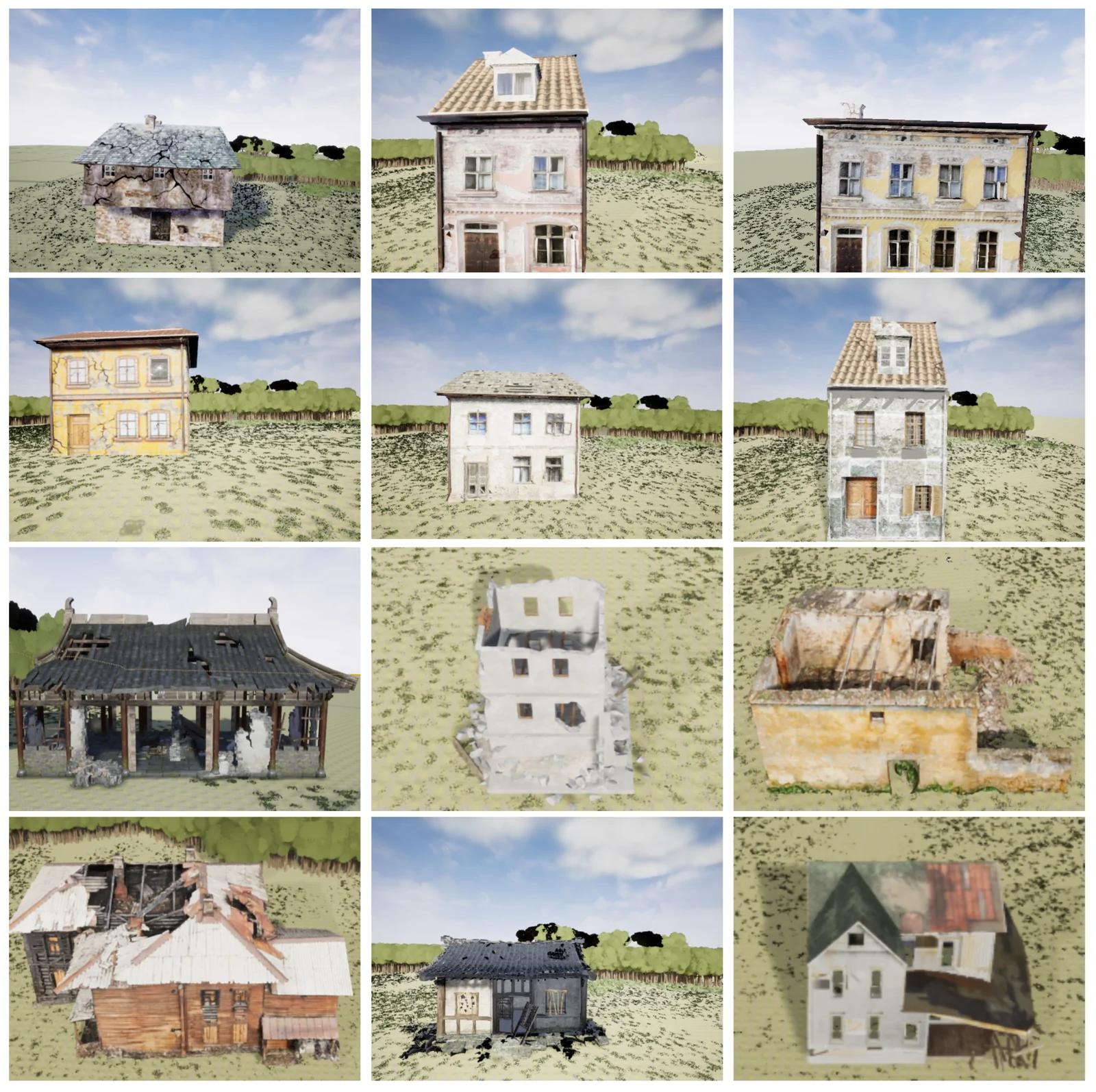
Dataset.

Our dataset was generated using AirSim [45]. AirSim provided the drone simulator. The environment was built in Unreal Engine 4. There are 12 scenes in the dataset, as shown in Figure 7. The first six buildings mainly simulate minor damage, such as wall cracks, peeling of wall coverings, and roof detachment. The last six buildings primarily simulate severe damage situations, such as the collapse of the support structure and walls. We invited 3D model designers to construct models based on images of actual damage. These simulated scenarios enable us to access various types of real-world environments that are difficult to obtain data from in a short period. In addition, we validated the proposed algorithm using data from reduced-scale models and the real explosion scene.

### 4.2. 3D Reconstruction

**Figure 8:**
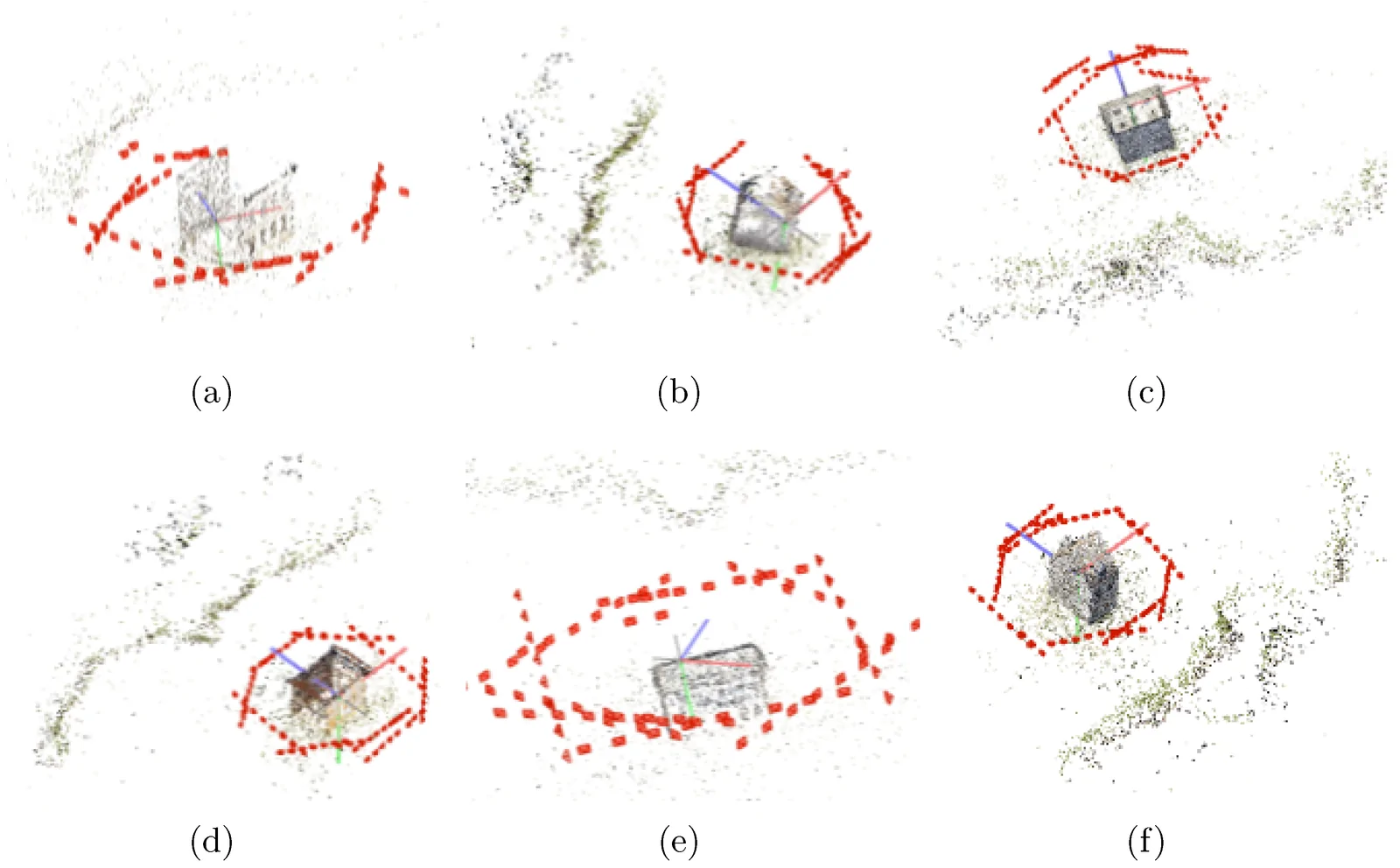
3D reconstruction results. (**a**–**f**) are reconstructed point clouds of different scenes. The red boxes represent the camera poses, and the other points are the reconstructed 3D points.

The 3D reconstruction results are obtained by structure from motion (SfM) [41]. The results are shown in Figure 8. The red points represent the camera poses, while the other points represent the reconstructed point cloud. We do not restrict the camera to be in the same position before and after the damage, but we only need to ensure that the outer surface of the building is captured. We do not require as many images as possible before the damage. Experiments show that more images and a higher density of reconstructed point clouds may lead to lower accuracy of point cloud registration, possibly due to accumulated reconstruction noise and redundant features.

### 4.3. Point Cloud Registration Results

**Figure 9:**
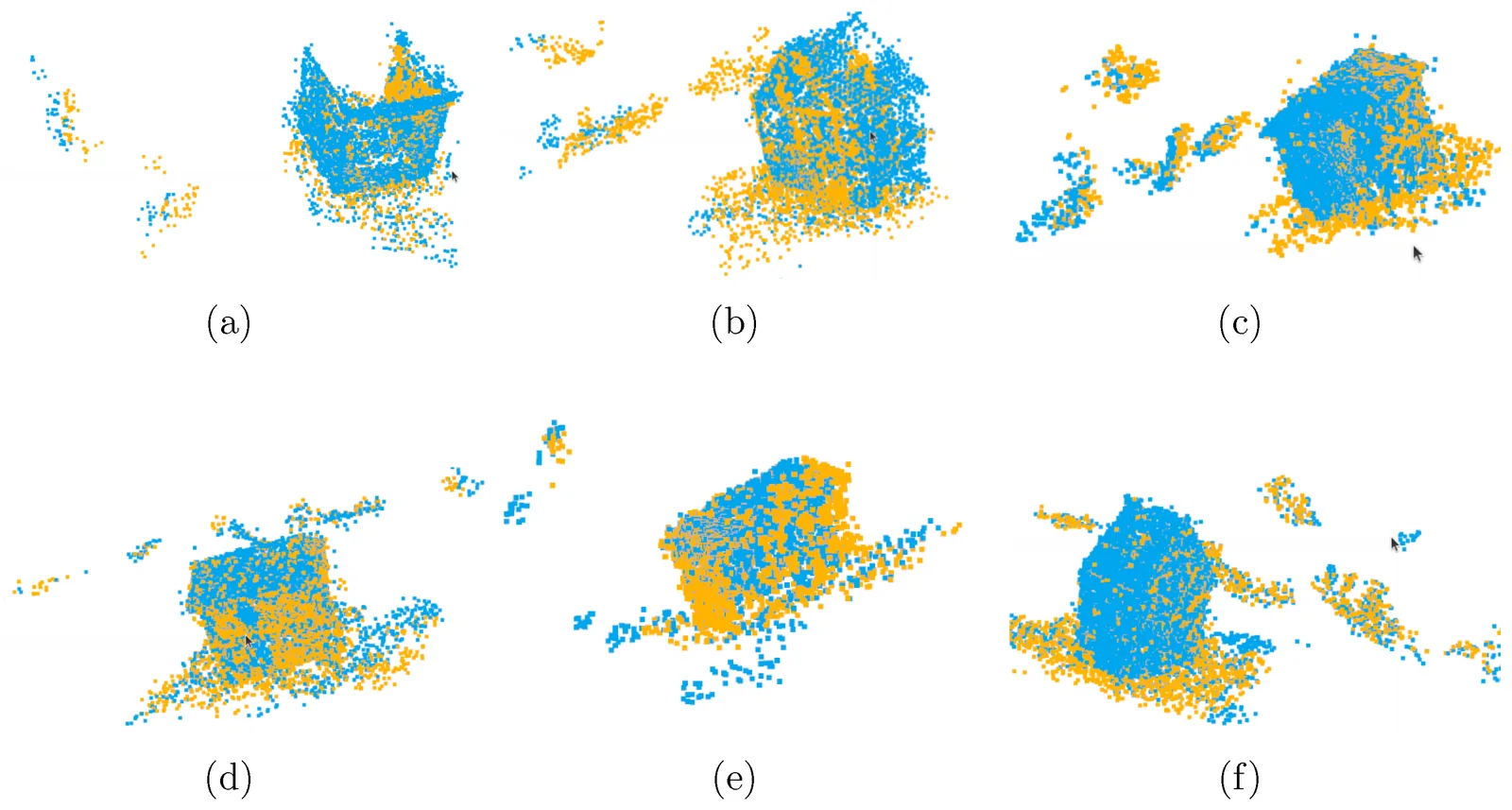
Point cloud registration results. (**a**–**f**) are point cloud registration results of different scenes. The yellow and blue dots represent the 3D points reconstructed before and after damage, respectively.

We implement our method with PyTorch 2.3.1 on an RTX 4090 GPU with an Intel CPU. We train the model separately with the Adam optimizer [46] for 40 epochs on our dataset and 100 epochs on KITTI. The batch size is set to 1, and the initial learning rate is $1\times 10^{-4}$ in the training stage. The point cloud registration results are shown in Figure 9. The yellow points and black points represent the pre-damage and post-damage point clouds, respectively. Experiments show that traditional point cloud registration methods can obtain results under certain constraints. We conduct a detailed analysis by collecting drone images from different altitude trajectories. Specifically, starting from the pre-damage flight altitude, data collection after the damage is carried out at intervals of 1 m, with an upward distance of 8 m and a downward distance of 7 m. After multiple point cloud registration experiments, it is found that good results can be achieved within a 2-m height difference in the simulation environment.

**Figure 10:**
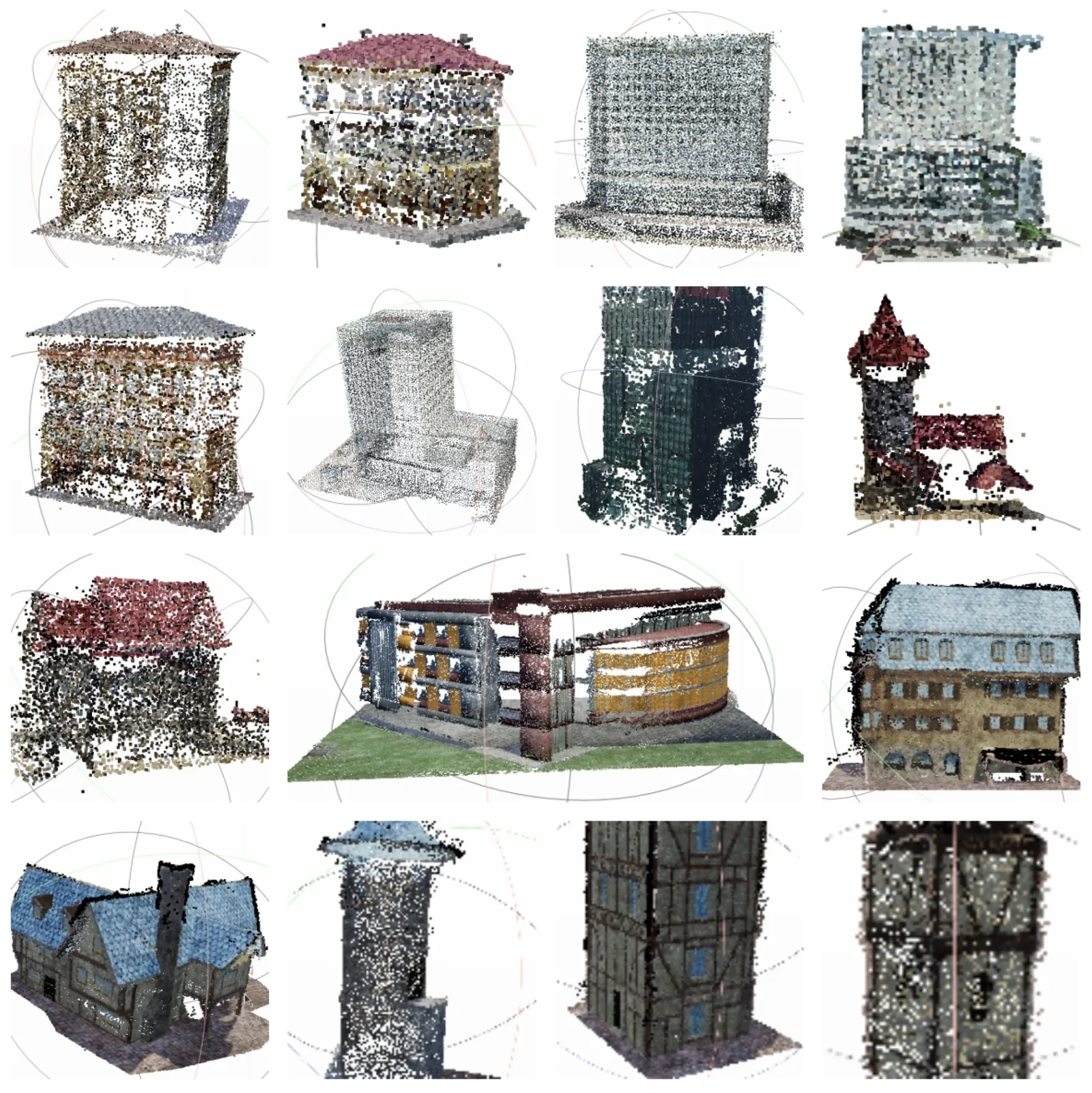
Training samples for point cloud registration.

The accuracy of point cloud registration directly determines the quality of the estimated spatial transformation and, consequently, the reliability of the entire damage assessment pipeline. Through experiments, we found that traditional registration methods have large errors at low overlap rates between the two models. Therefore, we constructed a point cloud registration dataset tailored to scenarios involving complex variation, as shown in Figure 10. The overlap rate between partial and complete point clouds in the dataset ranges from 20% to 50%. Furthermore, 60% of paired point clouds exhibit an overlap rate below 30%. The dataset for training was derived from UrbanScene3D [47], and the test dataset was our constructed data. We used 2660 pairs of point clouds to train the model and evaluated it on 200 pairs. We utilized relative rotation error (RRE) and relative translation error (RTE) following [48] to measure the differences between the predicted and the ground-truth matrices or translation vectors. These two metrics are shown in Equations (10) and (11).  

(10) $$RRE=arccos(\frac{trace(R^{T}\cdot \bar{R}-1)}{2})$$

(11) $$RTE=\|t-\bar{t}{\|}_{2}$$

The point cloud registration results are shown in Table 1. ICP [31], CoFiNet [35], GeoTrans [36], RoITr [49], MAC [37], Parenet [17], PTT [50], TEAR [51] and TurboReg [52] are compared methods used for point cloud registration. Our method achieved the minimum error on both RRE and RTE metrics. TurboReg [52] achieved the second-smallest errors in RRE and RTE. Our method effectively improves the accuracy of the baseline through a serialization superpoint matching module and reducing the training time. The proposed method achieved minimum error in both RRE (2.050) and RTE (0.291) metrics.The ablation experiments of key components are shown in Table 2. The experiment shows that our method takes approximately 6.5 h to train, compared to 8.5 h for the baseline method, resulting in a time savings of about 23%.

We verified the generalization performance of the proposed method on the KITTI Odometry dataset [48]. The urban dataset provides 11 sequences (00–10) with ground truth trajectories and LiDAR information. The data split for training, validation, and testing follows the protocol in [17]. In addition to the two metrics mentioned above, the evaluation protocol in the KITTI dataset also uses the registration recall metric to evaluate algorithm performance. Registration Recall (RR) is defined as the fraction of correctly registered point cloud pairs whose RRE and RTE satisfy the condition (i.e., RRE $<5^{{}^{\circ}}$ and RTE <2 m). As shown in Table 3, our method also achieves state-of-the-art results on the public outdoor dataset. The proposed method obtains the smallest error on the RRE metric and the highest score on the RR metric. By comparing the algorithm performance in Table 1, we found that the damage assessment dataset is significantly different from the daily outdoor dataset.

### 4.4. Image Generation

**Figure 11:**
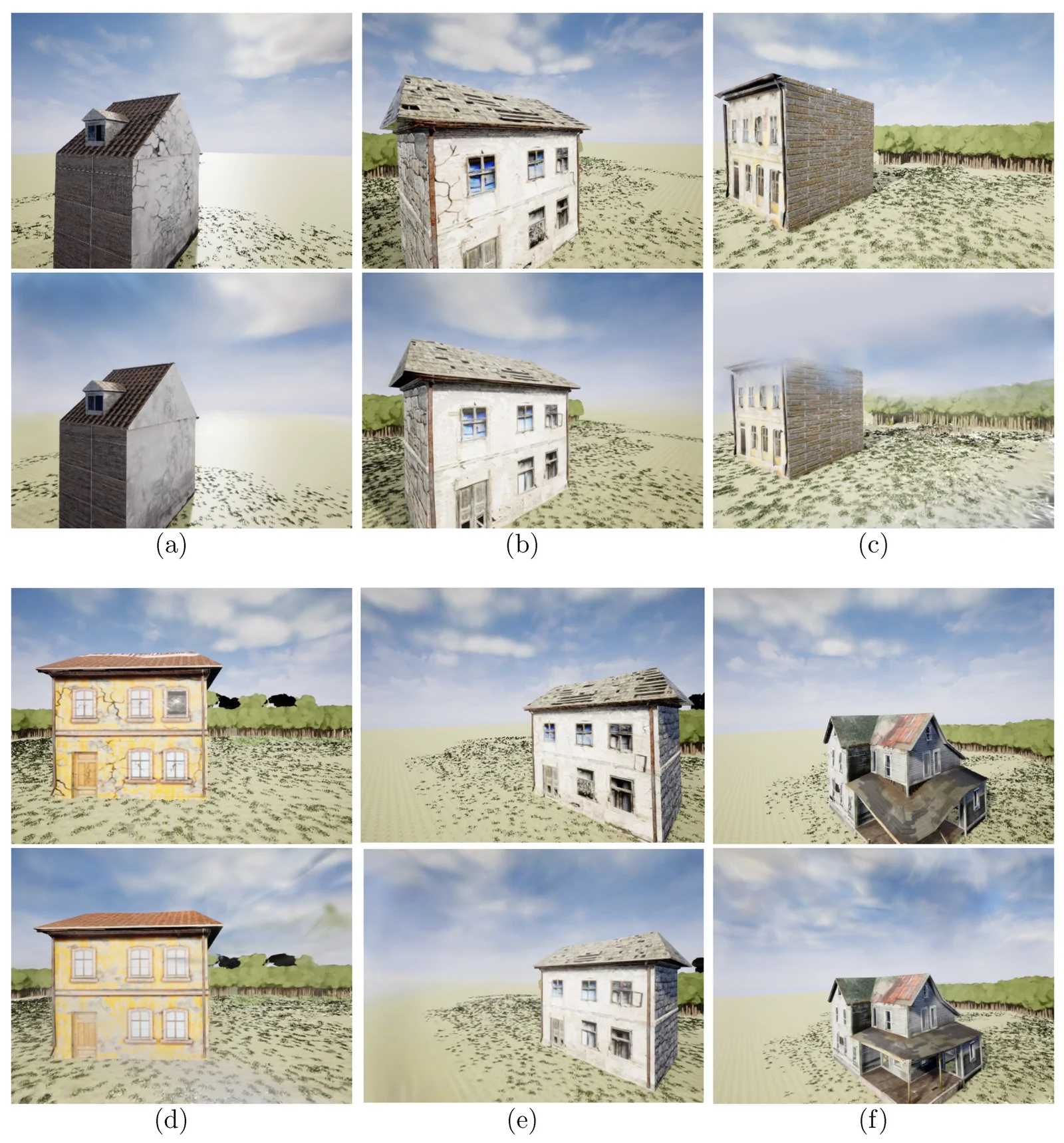
Image generation before the damage according to the pose of the drone after the damage. (**a**–**f**) represent several different scenes. In each subfigure, the top image is the post-damage image, and the bottom image is the generated pre-damage image from the same viewpoint.

We utilized learned perceptual image patch similarity (LPIPS) [54] and structural similarity index measure (SSIM) to evaluate our method’s performance. The ground truth consists of fixed-point images collected in AirSim. As shown in Table 4, the LPIPS metric values between the images we generated and the ground truth images range from 0.3 to 0.6. These quantitative results indicate that the images generated by the method appear visually similar to the ground-truth images when viewed by the human eye. According to our statistics, our method can reduce number of pre-damage images required by 1/2–2/3. In addition, we validated the effectiveness of the proposed method through qualitative experiments. The results are shown in Figure 11. By comparing the generated pre-damage images with the corresponding post-damage images from the same viewpoint, we can clearly identify various types of damage, including wall cracks, roof damage, wall collapses, and destruction of doors and windows.

### 4.5. Simulation Experiment for Damage Assessment

**Figure 12:**
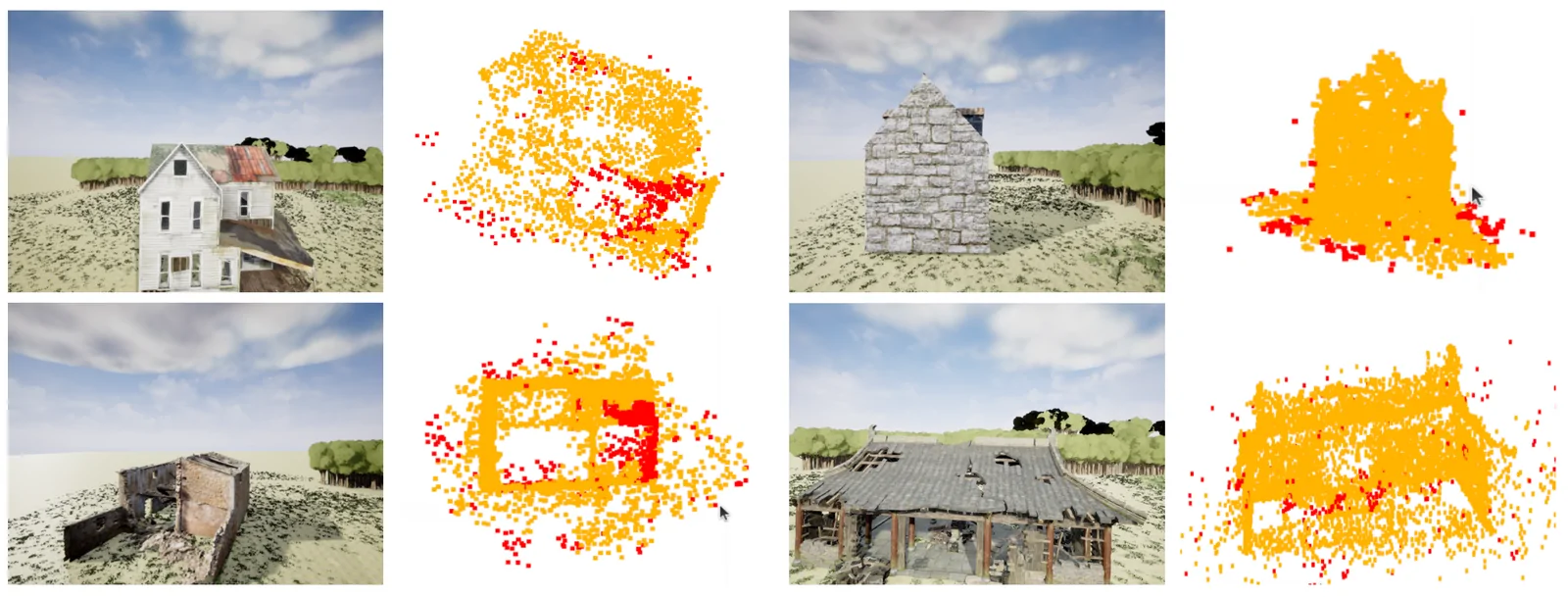
Damage assessment based on 3D point clouds. The first and third columns show post-damage scene images. The second and fourth columns show point cloud visualizations of the changed regions, with red points indicating the changed areas. The red dots represent the changed 3D points, and the yellow dots represent the unchanged 3D points.

**Figure 13:**
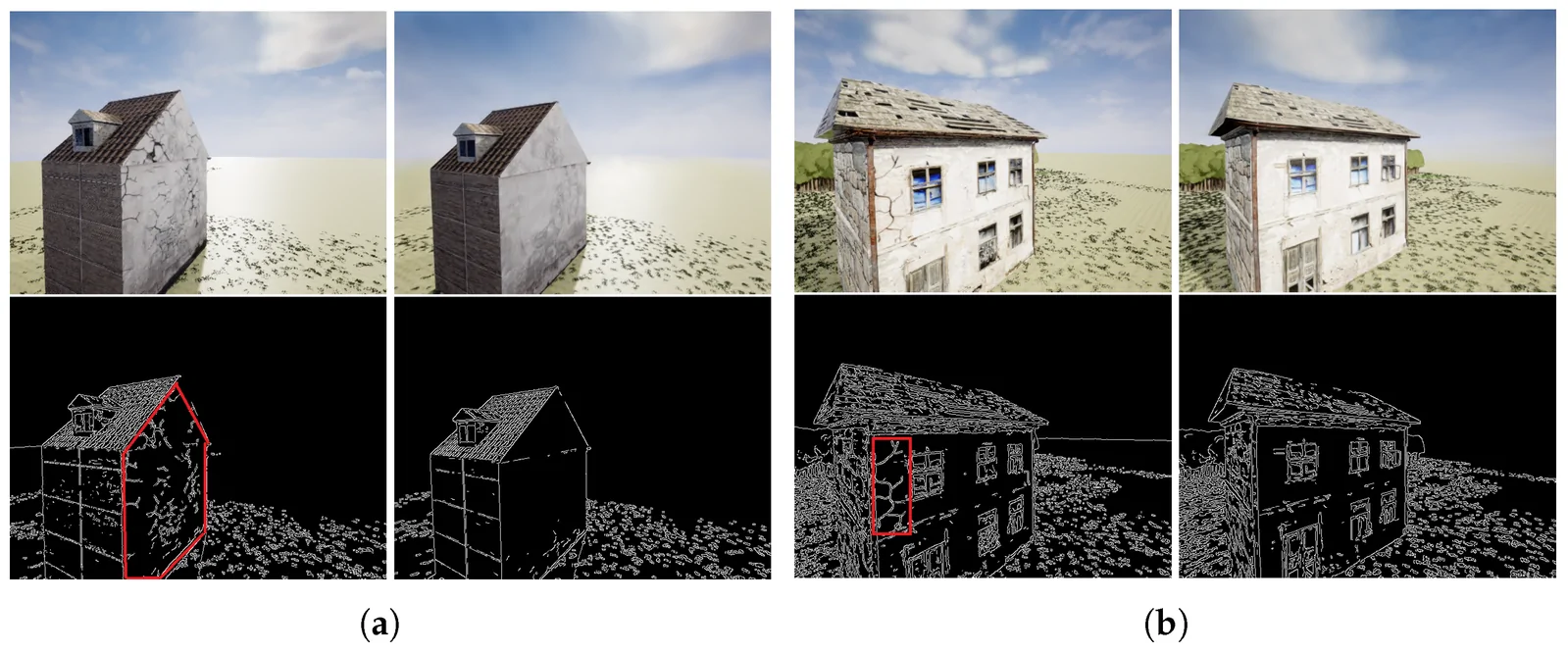
Damage assessment based on 2D images. (**a**) Example of damage on the side of a building. (**b**) Example of damage on the front of a building.The first and third columns show post-damage scene images. The second and fourth columns show pre-damage rendered images. The red boxes show the changed area.

**Figure 14:**
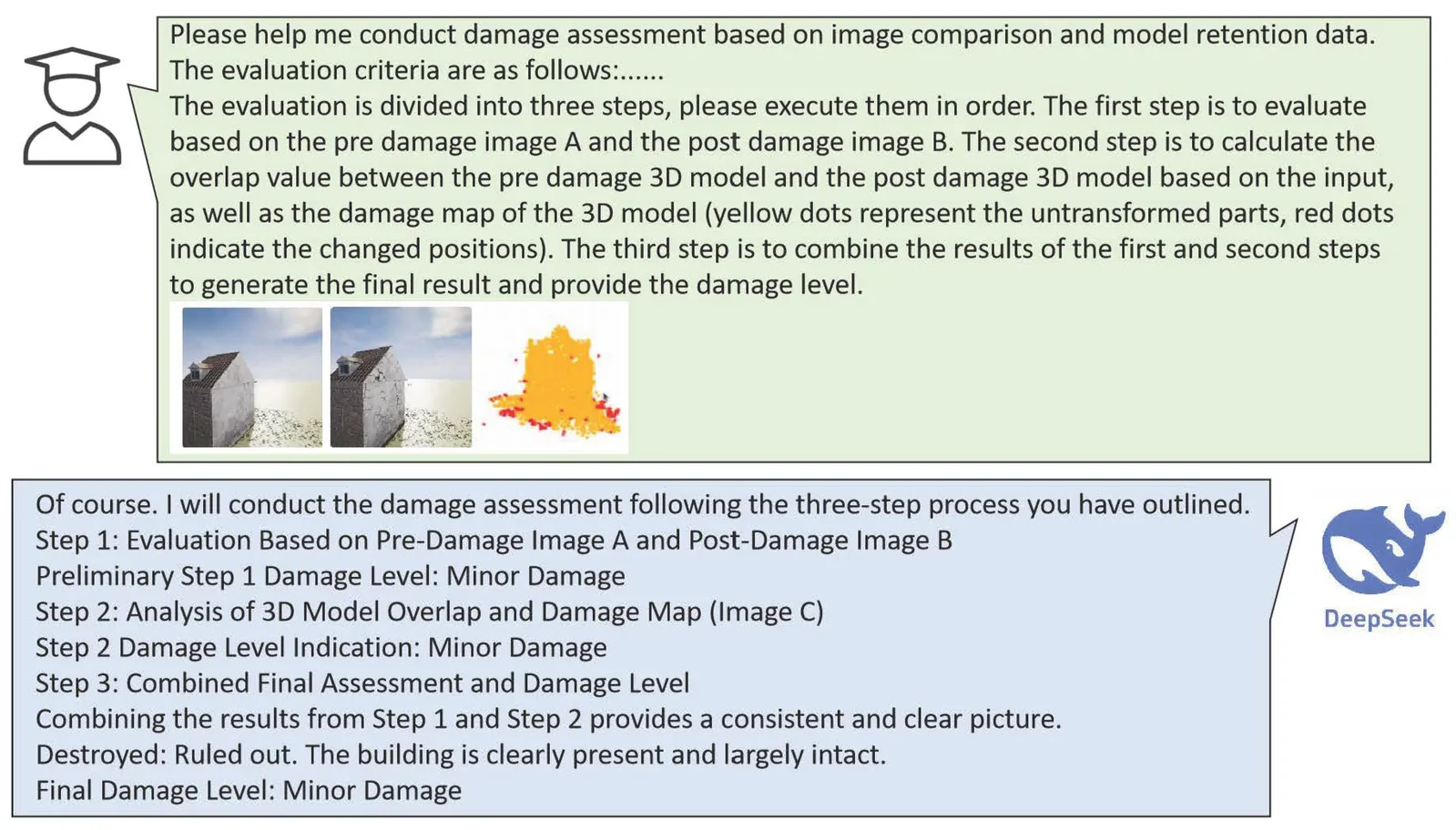
Damage assessment based on 2D images and 3D point clouds. The red dots represent the changed 3D points, and the yellow dots represent the unchanged 3D points.The assessment model is DeepSeek.

The 3D reconstruction and the corresponding 2D images can be used for damage assessment, as shown in Figure 12 and Figure 13. By registering the post-damage point cloud with the pre-damage point cloud, the structural damage to the building can be clearly visualized. The ratio of the overlapping point cloud to the source point cloud is calculated, as shown in Table 5. The overlap ratios of the first six scenes range from 0.8616 to 0.9694, while the others are shown in Table 5. This result verifies that our method can accurately reflect the changes made to the 3D model. Figure 14 illustrates an example of damage assessment using prompt words and multi-modal data. We provide DeepSeek with 3D model variation information via registration overlap rate and heatmaps, generating assessment results consistent with expectations. The prompt words are listed below, as shown in Listing 1.

**Listing 1.** Prompt for damage assessment. Author: We have cited this listing in the text.
Please assist me to conduct damage assessment based on image comparison and model retention data. The evaluation criteria are as follows:

No damage: … Minor damage: …

Major damage: … Destroyed:…

The evaluation is divided into three steps, please execute them in order. The first step is to evaluate based on the pre-damage image A and the post-damage image B. The second step is to calculate the overlap value between the pre-damage 3D model and the post-damage 3D model based on the input, as well as the damage map of the 3D model (yellow dots represent the untransformed parts, red dots indicate the changed positions). The third step is to combine the results of the first and second steps to generate the final result.


### 4.6. Reduced-Scale Model Experiment for Damage Assessment

#### 4.6.1. Case Study

**Figure 15:**
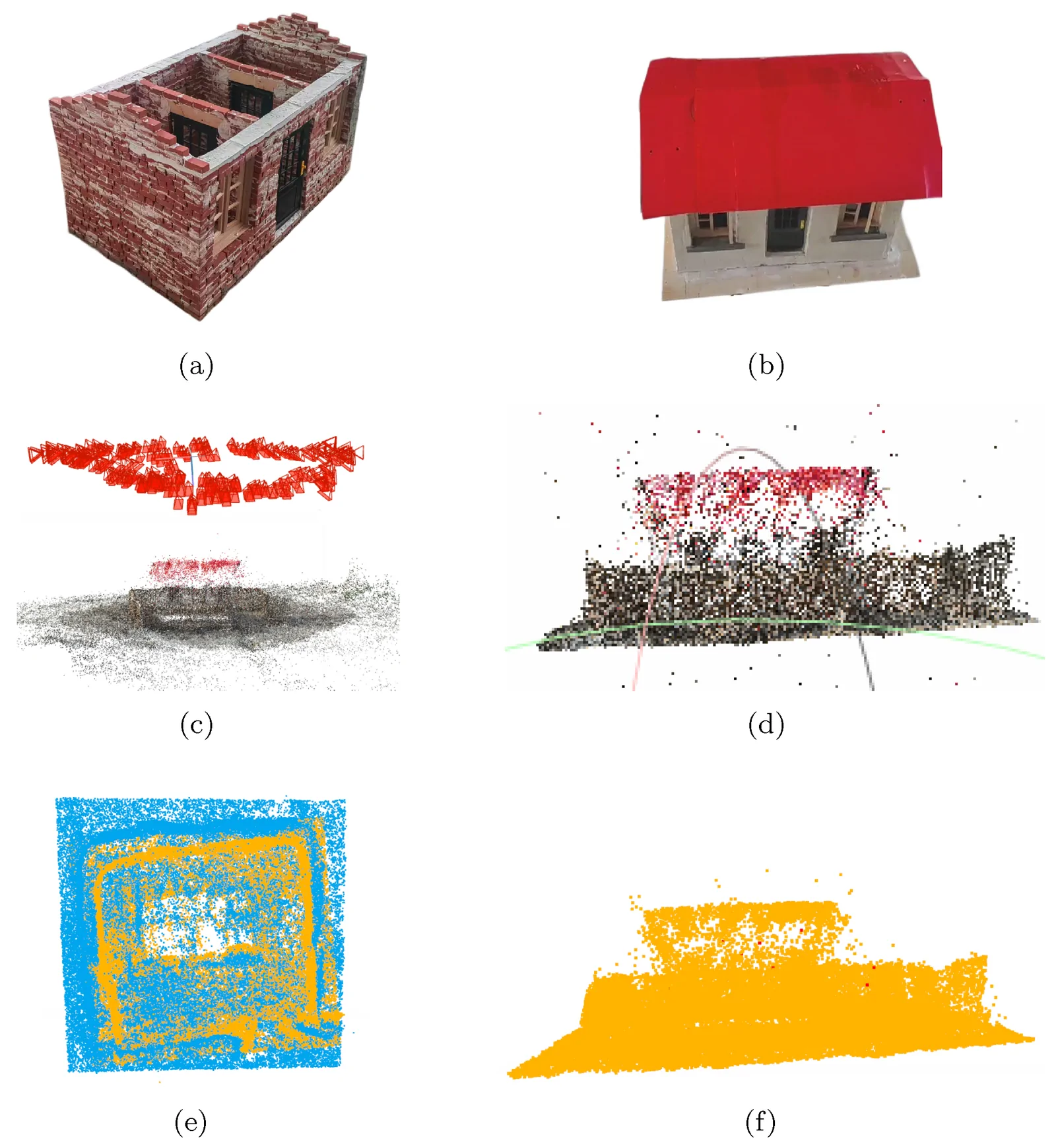
Damage assessment of reduced-scale model based on 3D point clouds. (**a**) Internal structure of the scaled model. (**b**) External structure of the scaled model. (**c**) Pre-damage point cloud. (**d**) Post-damage point cloud. (**e**) Registration result of the pre- and post-damage point clouds. (**f**) Visualization of the changed regions in the point cloud. The red dots represent the changed 3D points, and the yellow dots represent the unchanged 3D points.

In addition to the simulation tests described above, we also fabricated a scale model to simulate the damage assessment of buildings. The model was built at a 1:10 scale, based on a single-story house in a northern rural village. The scale model was designed based on the layout of a typical rural two-bedroom, one-living-room home and constructed using brick and tile, as shown in Figure 15a,b. We placed the model on a flat, open outdoor area and installed a fence around it. The problem we faced in the scaled model is that there are too many noise points in the outdoor scene. Figure 15c,d show the results of the three-dimensional reconstruction of the scale model before and after damage. The cracked soil and surrounding tree textures introduce a large number of noise points. Before applying the point cloud registration method, we separated the main models of the houses that needed to be registered through segmentation methods. The point cloud registration result is shown in Figure 15e. As shown in Figure 15f, the yellow points represent point clouds that remain consistent before and after damage, while the red points represent point clouds that have changed. The model did not undergo significant changes.

**Figure 16:**
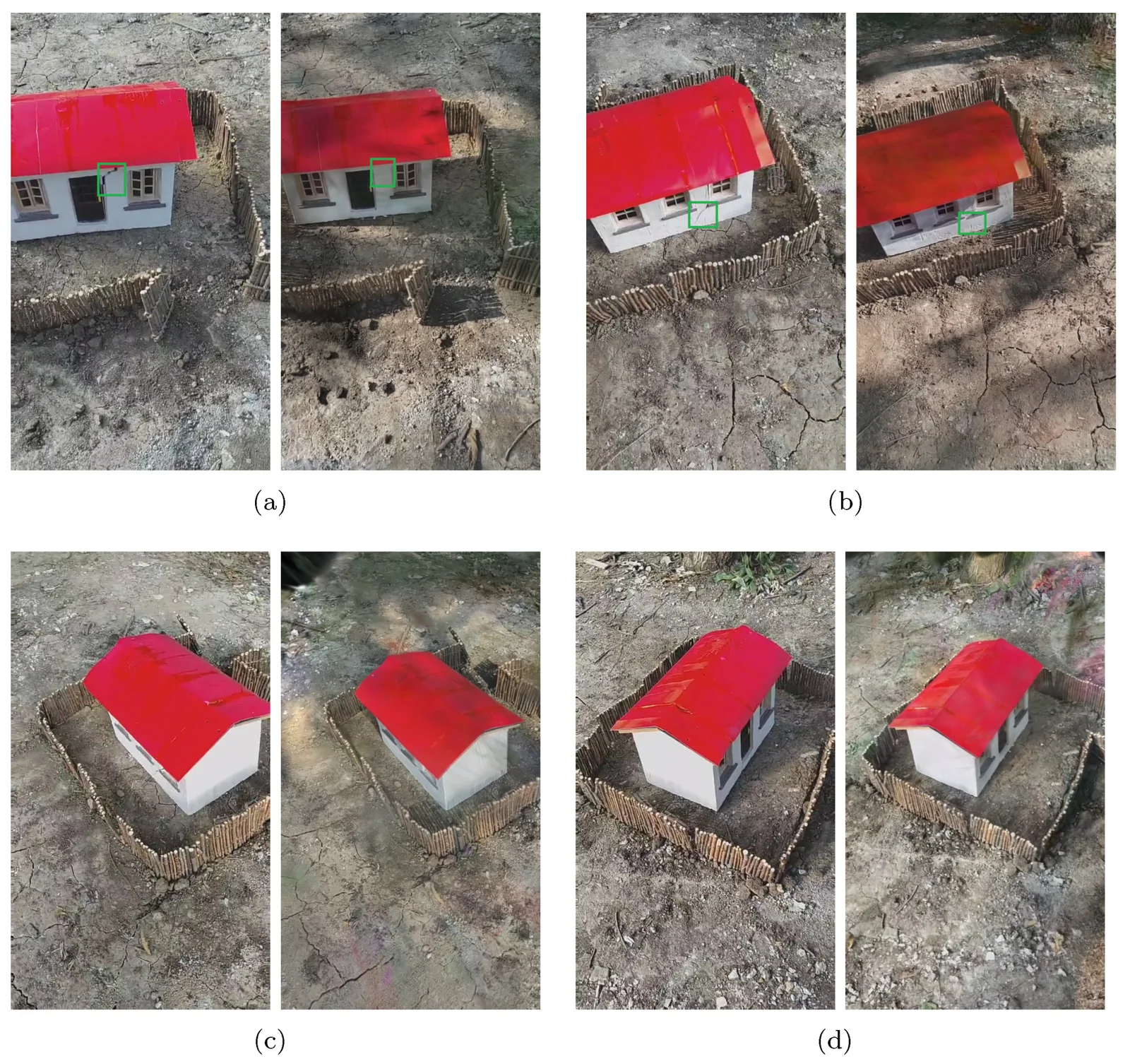
Damage assessment of the reduced-scale model based on 2D rendered images. Each subfigure compares the pre- and post-damage states. (**a**) Front view. (**b**) Back view. (**c**) Left side wall. (**d**) Right side wall. The green boxes represent the regions of interest.

Figure 16 shows images captured after the damage occurred and pre-damage rendered images using the proposed method based on the pose of the post-damage image. Within all the sub-figures, the image on the left is a real photograph, and the one on the right is a rendered image. By comparing each subgraph, we found that the quality of the rendered image was high and matched well with the damaged image. By analyzing the above comparison pictures, the conclusion reached by the LLM model is that the main structure of the building is intact, with a high overall point cloud overlap rate and no significant changes observed. Specifically, there are cracks in the walls and physical structural alterations beneath the eaves, which constitute minor damage.

#### 4.6.2. Ablation Experiments on Multimodal Data

**Figure 17:**
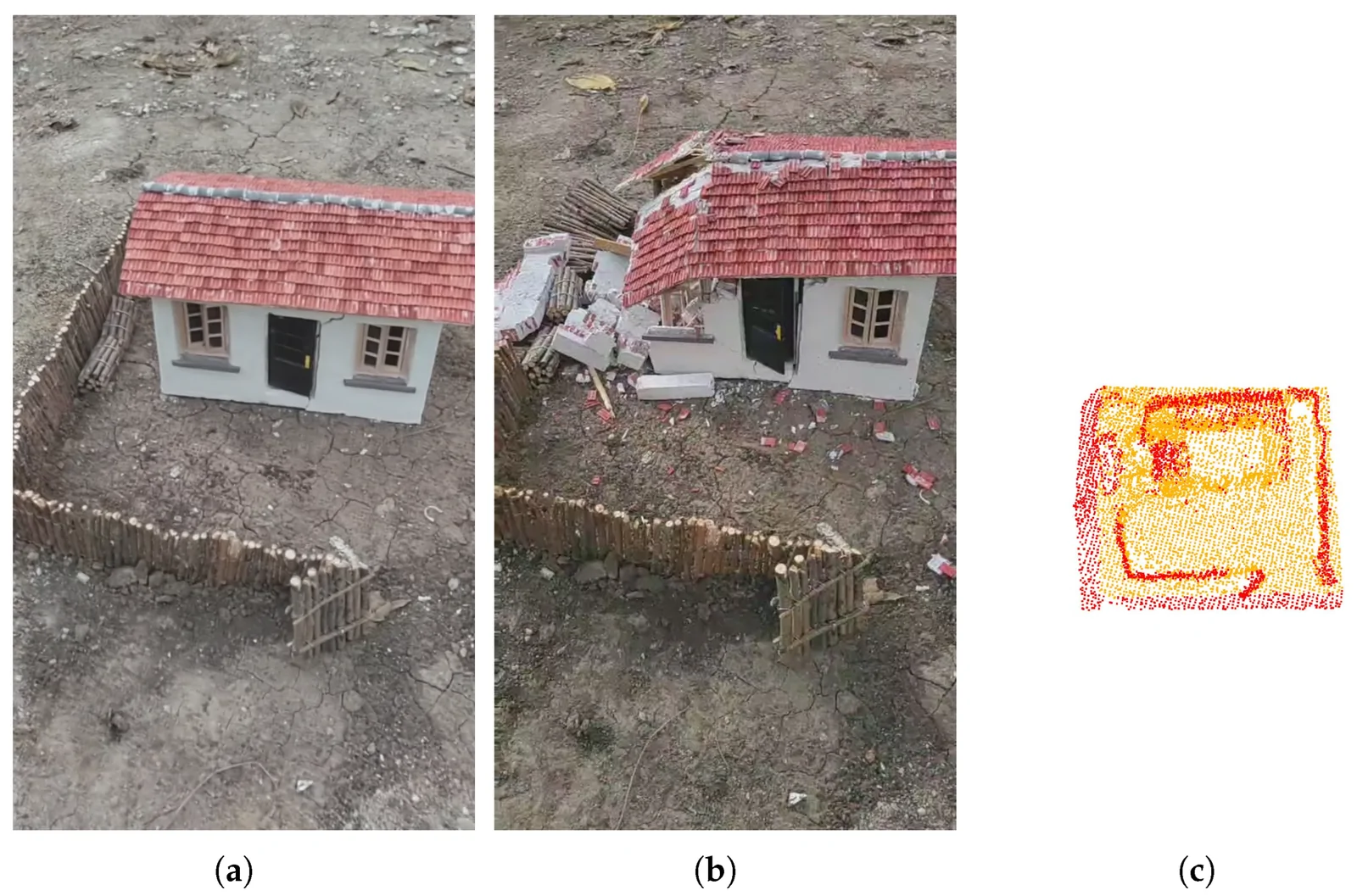
Ablation study of damage assessment. (**a**) Pre-damage image. (**b**) Post-damage image. (**c**) Visualization of changed regions in the point cloud. The red dots represent the changed 3D point, and the yellow dots represent the unchanged 3D points.

We conducted ablation experiments on the impact of different modalities by collecting data in severely damaged scenarios. We further refined the scale model. First, we used scaled-down tiles to create the roof, simulating the realistic effect of tiles falling and breaking. Second, we knocked down one side wall and scattered the bricks nearby. As shown in Figure 17a,b are used solely as inputs for 2D damage assessment. Figure 17c and the overlap ratio are used solely as inputs for 3D damage assessment. Finally, we used images (a–c), and the overlap ratio as inputs for our proposed method. Our prompt is shown in the Table 6. These three ablation experiments used the same damage assessment criteria. Due to space limitations, we have omitted the step-by-step results of the LLM in the main text and listed only the main conclusions, as shown in Table 6. By analyzing the ablation experiments, we found that significant damage results can be obtained through either 2D or 3D evaluation alone; however, the 2D evaluation conclusions contained an erroneous description of “ground cracking.” Nevertheless, combining the results of 2D and 3D evaluations could avoid the “ground cracking” issue. In terms of quantitative analysis, 2D evaluation relied on two criteria; one of them was incorrect, yielding an accuracy of 50%. 3D evaluation relied on three criteria, achieving an accuracy rate of 100%. The combined 2D+3D evaluation relied on three criteria, with an accuracy rate of 100%. The conclusion of the damage assessment in this scenario is major damage.

#### 4.6.3. Robustness Experiment

To verify the superiority of the proposed damage assessment method based on 3D registration, we further conducted robustness experiments using a scaled-down model to assess the method’s performance under conditions of motion blur in the images. First, we performed 2D registration on three image datasets: pre-damage, post-damage, and pre- and post-damage pairs. As shown in the first column of Table 7, 2D registration achieved an average success rate of 26.6% in the undamaged dataset. However, the success rate of 2D registration in the data after damage was only 7.4%. The success rate of 2D image registration tasks before and after damage decreased to 2.3%. The above experiment indicated that the 2D registration method is not suitable for pre- and post-damage registration. However, exhaustive pairwise registration is computationally expensive. By extracting 10% of the images for sampling, as shown in the second column of Table 7, the overall trends were preserved.

In addition, we evaluated the performance of 2D and 3D registration methods under the motion blur condition by randomly introducing motion-blurred images at proportions of 10–50% into the dataset. As shown in Table 8, in the registration task between pre-damaged and post-damaged images, as the proportion of motion-blurred images increased, the registration accuracy and inlier ratio of the registration method gradually decreased. When the proportion of blurred images reached 50%, the success rate decreased by 47.8% compared to the baseline, and the proportion of inliers decreased by 35.8% relative to the baseline. As shown in Table 9, for pre- and post-damage point cloud registration, there was only a slight decrease in the point cloud overlap rate (0.4–5.5%).

### 4.7. Real-World Experiment for Damage Assessment

Our method can also be applied to damage assessment of explosion accidents. Videos are captured by a GoPro 11 camera from [55,56]. When applying the method to real explosion experiments, we faced two problems: one is that the fisheye lens of the GoPro 11 caused severe deformation of the edges of the house, and the other is that the resulting image resolution exceeded the GPU memory capacity. Edge deformation led to feature matching errors in 3D sparse reconstruction of houses, resulting in distortion and severe deformation of the reconstructed houses. Excessive image resolution caused GPU memory overflow during model rendering. Therefore, in practical applications, this method requires two additional steps: one is to remove distortion from the image, and the other is to proportionally reduce the image. We used FFmpeg software with the lenscorrection filter to eliminate distortion in images captured by fisheye lenses.

**Figure 18:**
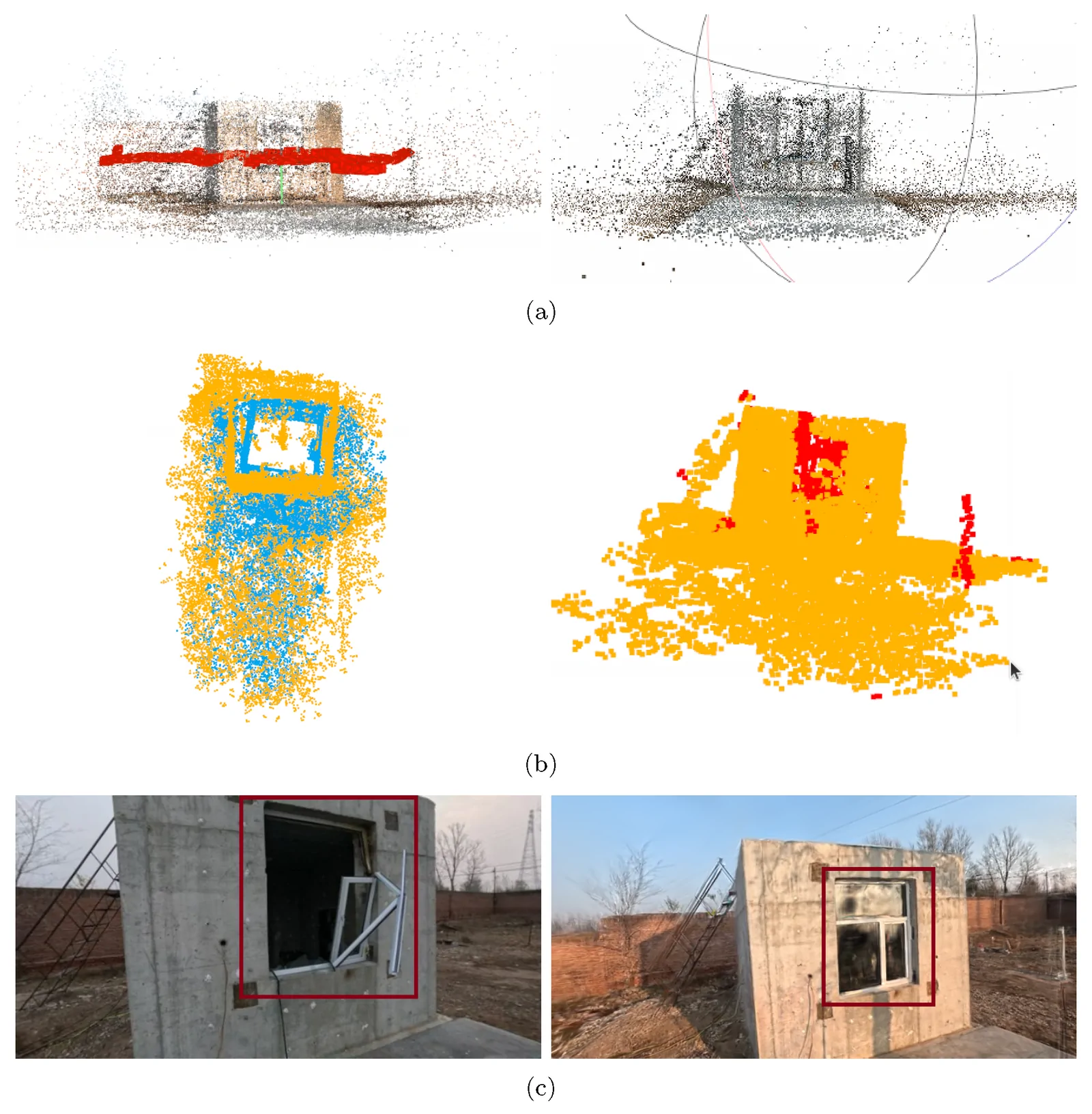

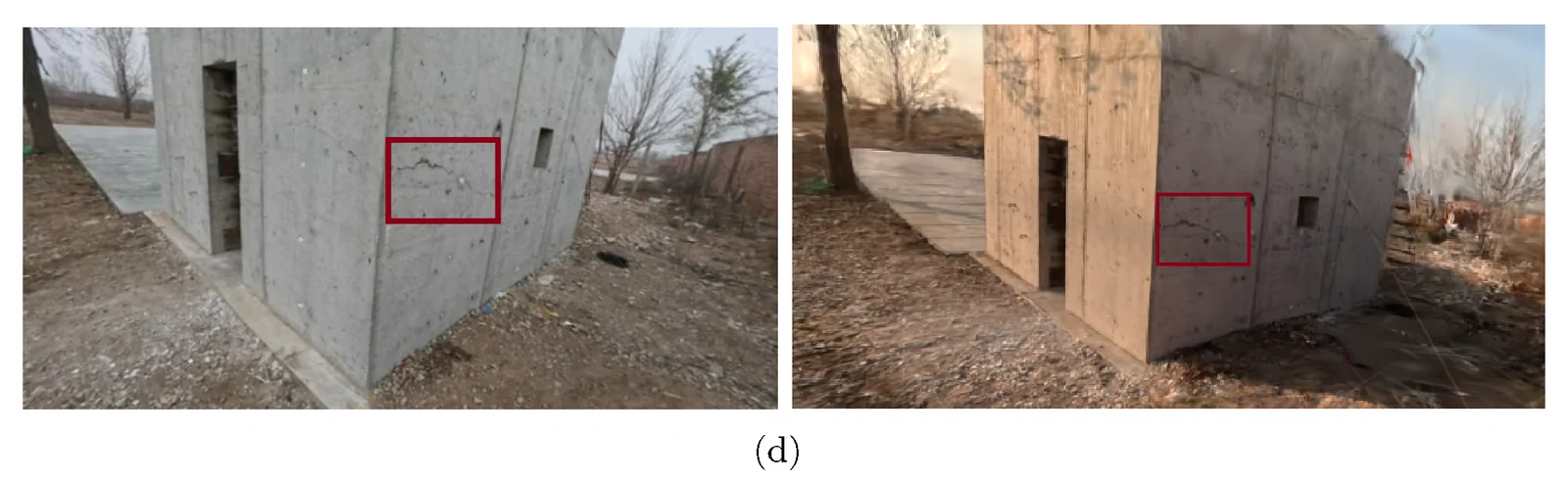
Damage assessment of real explosive buildings. (**a**) Pre- and post-damage point clouds. (**b**) Registration results and change visualization of the point clouds. The dots in (**a**,**b**) represent the 3D points. The red dots represent the changed 3D points. (**c**) Front view of the building before and after damage. (**d**) Back view of the building before and after damage. The boxes in (**c**,**d**) highlight the regions of interest.

The 3D reconstruction results are shown in Figure 18a. The point cloud registration result is shown in Figure 18b. Figure 18c,d are comparative images of damage assessment from different perspectives. The left side is the image captured after damage, and the right side is the image generated by rendering the model before damage. By analyzing the above images, the LLM model gives an automated and accurate assessment. The window frame is severely damaged. The white plastic steel or aluminum alloy window frame has completely detached from the window opening and has undergone severe twisting, breaking, and folding. The window frame has lost its original geometric shape and supporting function. The comparison between these two red boxes indicates that the explosion damaged the windows but did not cause structural damage to the concrete walls. The damage assessment results from the actual explosion scenario indicated minor damage.

## 5. Discussion

Our proposed method can be applied to different damage assessment frameworks by quantifying the damage results. The definition of damage level varies from country to country, and it can be categorized into three, four, or five categories. Therefore, in this paper, we do not directly correlate images with damage levels.

**Figure 19:**
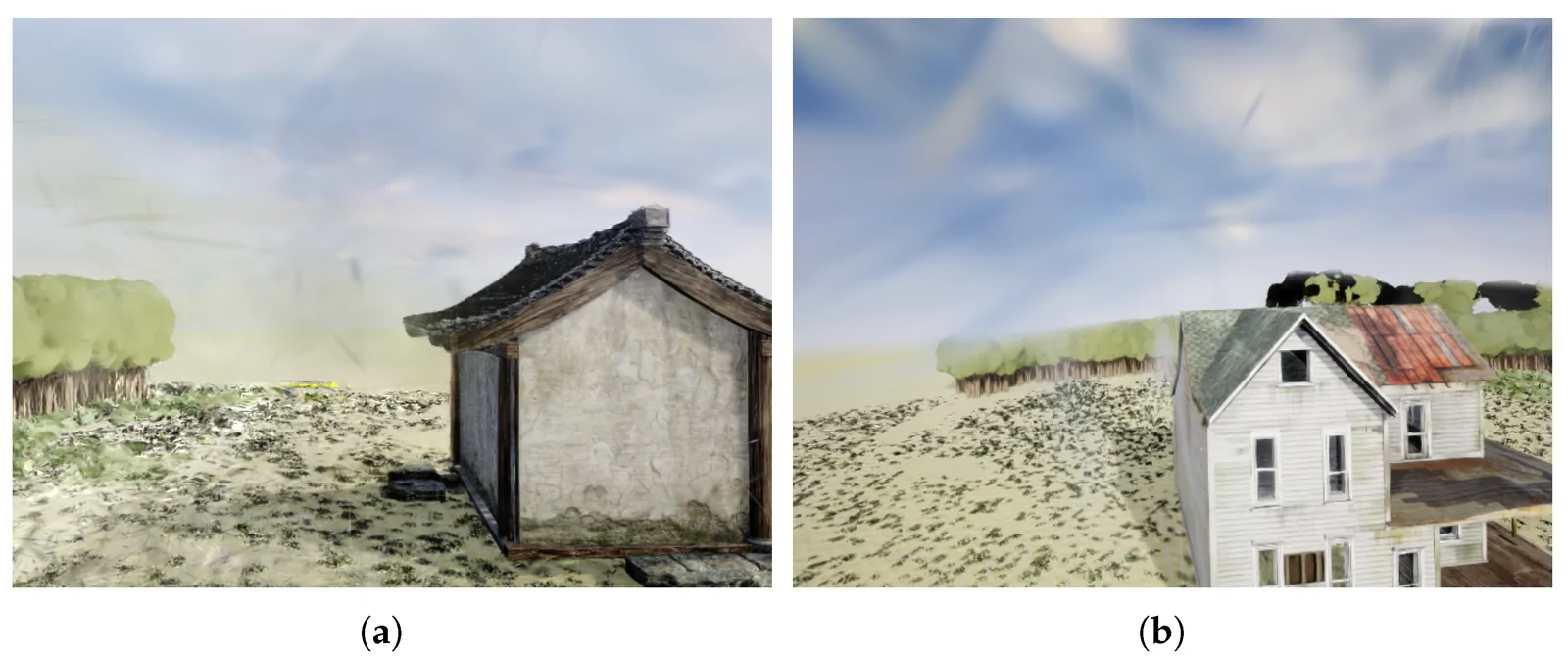
Failure cases. (**a**) Rendered image of a traditional building with clear architecture but blurred sky artifacts. (**b**) Rendered image of a two-story house with similar blurred-sky artifacts.

Through experiments, we found that if the height difference between the perspective of the image taken before the disaster and the perspective of the image captured after the disaster is small, a relatively ideal image can be generated. However, if the height difference is too large, it is still impossible to generate images from an unseen perspective. As shown in Figure 19, If the camera was positioned relatively low during pre-disaster image capture, the rendered background (e.g., the sky) appears blurry, while the main structure remains clear. However, with the rapid development of the low-altitude economy, an increasing number of drones are being deployed for structural health monitoring tasks, and the volume of pre-disaster building imagery is growing significantly. In addition, we can further expand the application scope of the proposed method by combining the point cloud with building information model (BIM). BIM contains 3D models constructed through architectural drawings. By sampling the 3D geometric model of BIM, the point cloud before destruction can be obtained. By importing a 3D geometric model into the UE environment and using the AirSim plugin, images can be used to train models. As shown in [57] and our paper, post-damage point cloud be obtained by cluster or segmentation algorithm. The alignment process can be performed using either traditional or deep-learning-based methods.

Our method relies on the accuracy of point cloud registration. The performance of the registration method is related to the input point cloud. If we aim to obtain the most comprehensive pre-damage model possible, we should take as many images as possible from various angles. More images generally mean a denser point cloud. However, the time available for data acquisition after the damage is limited, and the number of images obtained is limited. There is a significant registration error between dense point clouds and sparse point clouds. Preprocessing methods such as downsampling can improve the robustness of the method.

## 6. Conclusions

A timely assessment of damaged buildings is critical for subsequent decision-making. We propose a method of assessing building damage based on images captured by unmanned aerial vehicles (UAVs). This method eliminates the need for paired images from the same perspective obtained through traditional photography methods, thereby reducing the difficulty and cost of data collection. Our method can effectively support the task of damage assessment. We not only use two-dimensional images from the same perspective before and after damage for assessment but also utilize the three-dimensional structural differences before and after damage as assessment indicators to enhance accuracy. Our method uses point cloud registration based on point cloud serialization to perform spatial transformations, overcoming the limitations of two-dimensional image registration methods, which are affected by factors such as lighting, occlusion, and blurring. This enhances adaptability and improves the efficiency of damage assessment further.

## Figures and Tables

**Table 1:** Results of point cloud registration under conditions of damage assessments. The year of a combined method is based on the most recent method in the combination. The downward arrow (↓) indicates that a lower value is better.

Method	RRE↓	RTE↓	Year
FPFH [30] + ICP [31]	110.583	22.059	2009
CoFiNet [35]	61.628	30.734	2021
GeoTrans [36]	3.422	0.841	2022
RoITr [49]	63.560	30.737	2023
FPFH [30] + MAC [37]	74.467	32.788	2024
Parenet [17]	5.813	1.275	2024
TEAR [51]	89.92	20.68	2024
PTT [50]	63.35	15.36	2025
TurboReg [52]	3.319	0.719	2025
Ours	2.050	0.291	2026

**Table 2:** Ablation study of point cloud registration under conditions of damage assessments. The downward arrow (↓) indicates that a lower value is better.

Method	RRE↓	RTE↓	Training Time
Vanilla Transformer	5.115	0.556	∼6 h
GeoTrans [36]	3.422	0.841	∼8.5 h
SerializeTransformer (Ours)	2.050	0.291	∼6.5 h

**Table 3:** Results on KITTI Odometry. The upward arrow (↑) indicates that a higher value is better, and the downward arrow (↓) indicates that a lower value is better.

Method	RRE (°)↓	RTE (cm)↓	RR (%)↑	Year
FCGF [53]	0.30	9.5	96.6	2019
CoFiNet [35]	0.41	8.2	99.8	2021
GeoTrans [36]	0.24	6.8	99.8	2022
MAC [37]	0.35	7.82	98.9	2024
Parenet [17]	0.24	5.3	99.8	2024
TEAR [51]	0.39	8.62	99.1	2024
PTT [50]	0.23	6.4	99.8	2025
TurboReg [52]	0.40	8.12	98.38	2025
Ours	0.23	6.4	99.8	2026

**Table 4:** Image generation results.The upward arrow (↑) indicates that a higher value is better, and the downward arrow (↓) indicates that a lower value is better.

Scene	SSIM↑	LPIPS↓	Scene	SSIM↑	LPIPS↓
House1	0.556 ± 0.051	0.467 ± 0.058	House7	0.574 ± 0.038	0.412 ± 0.028
House2	0.654 ± 0.057	0.404 ± 0.073	House8	0.459 ± 0.041	0.526 ± 0.036
House3	0.564 ± 0.068	0.379 ± 0.029	House9	0.567 ± 0.096	0.469 ± 0.054
House4	0.584 ± 0.057	0.355 ± 0.050	House10	0.638 ± 0.057	0.392 ± 0.022
House5	0.519 ± 0.048	0.515 ± 0.030	House11	0.570 ± 0.039	0.402 ± 0.035
House6	0.548 ± 0.044	0.402 ± 0.059	House12	0.463 ± 0.058	0.574 ± 0.048

**Table 5:** Damage assessment based on multimodal data.

Scene	Overlap Ratio	Assessment
House1–6	0.8616–0.9694	The main structure is complete. There are cracks on the exterior walls of the building.
House7	0.765	The main structure of one room has collapsed, and the roof of another room has fallen off.
House8	0.7687	The external facade of the room collapsed.
House9	0.7915	There are two damaged areas on the roof of the building, one accompanied by collapse and the other with minor damage.
House10	0.8352	The main structure of the building appears to be undamaged, but the roof of the wooden corridor has collapsed on one side.
House11	0.9639	Through 3D model analysis, the damage was mainly concentrated on the outer surface.
House12	0.6454	The roof tiles are largely missing or severely displaced, exposing the underlying wooden structure.

**Table 6:** Ablation study of damage assessment based on multimodal data. The ✔ indicates that the criterion is correct, and the ✗ indicates that the criterion is incorrect.

Module	Item	Assessment
2D	Prompt	Please evaluate based on the pre-damage image A and the post-damage image B.
	Result	Major damage. The structure has experienced a partial roof collapse (✔) and significant ground disturbance (✗), meeting the criteria for major damage.
3D	Prompt	Please evaluate based on the overlap value between the pre-damage 3D model and the post-damage 3D model based on the input, as well as the damage map of the 3D model (yellow dots represent the untransformed parts, red dots indicate the changed positions).
	Result	Major Damage. The 3D data confirms that while the main structure is still present (✔), nearly one-third of the model has transformed (✔), with the damage map highlighting a severe localized disruption in the roof/perimeter area consistent with a partial collapse (✔).
3D + 2D	Prompt	The evaluation is divided into three steps, please execute them in order. The first step is to evaluate based on the pre-damage image A and the post-damage image B. The second step is to calculate the overlap value between the pre-damage 3D model and the post-damage 3D model based on the input, as well as the damage map of the 3D model (yellow dots represent the untransformed parts, red dots indicate the changed positions). The third step is to combine the results of the first and second steps to generate the final result and provide the damage level.
	Result	Major Damage The structure has suffered Major Damage. While the main walls are still standing (✔), the partial collapse of the roof and the substantial structural changes indicated by the 3D overlap value (31.5% altered (✔) and damage map confirm this classification (✔).

**Table 7:** 2D registration result between different dataset.

Dataset	Registration Ratio	Registration Ratio
Pre-damage Dataset	0.266 ± 0.093	0.288 ± 0.096
Post-damage Dataset	0.074 ± 0.045	0.070 ± 0.037
Pre-Post Dataset	0.023 ± 0.027	0.024 ± 0.026

**Table 8:** Performance of the 2D registration method under different proportions of motion-blurred images.

The Degree of Ambiguity	Registration Ratio	Inlier Ratio
None (base)	0.023 ± 0.026	0.151 ± 0.021
10%	0.021 ± 0.024 (−8.7%)	0.141 ± 0.021 (−6.6%)
20%	0.019 ± 0.021 (−17.3%)	0.130 ± 0.020 (−13.9%)
30%	0.016 ± 0.017 (−30.4%)	0.114 ± 0.019 (−24.5%)
40%	0.014 ± 0.015 (−39.1%)	0.108 ± 0.018 (−28.5%)
50%	0.012 ± 0.014 (−47.8%)	0.097 ± 0.019 (−35.8%)

**Table 9:** Performance of the 3D registration method under different proportions of motion-blurred images.

The Degree of Ambiguity	Overlap Ratio	Inlier Ratio
None (base)	0.685	0.233
10%	0.681 (−0.4%)	0.184 (−21.0%)
20%	0.647 (−5.5%)	0.177 (−24.0%)
30%	0.673 (−1.8%)	0.179 (−23.2%)
40%	0.679 (−0.8%)	0.177 (−24.0%)
50%	0.672 (−1.8%)	0.176 (−24.5%)

## Data Availability

Data will be made available upon request. The videos of the explosion scene are sourced from [55,56].
